# Estimation of the Reliability of a Stress–Strength System from Poisson Half Logistic Distribution

**DOI:** 10.3390/e22111307

**Published:** 2020-11-17

**Authors:** Isyaku Muhammad, Xingang Wang, Changyou Li, Mingming Yan, Miaoxin Chang

**Affiliations:** 1 School of Mechanical Engineering and Automation, Northeastern University, Shenyang 110819, China; isyakuedu@yahoo.com (I.M.); david.yan0086@gmail.com (M.Y.); changmiaoxin@stumail.neu.edu.cn (M.C.); 2 College of Mechanical and Electrical Engineering, Guangdong University of Petrochemical Technology, Maoming 525000, China; 3 School of Control and Engineering, Northeastern University, Qinhunangdao 066004, China

**Keywords:** Poisson half logistic, stress-strength parameter analysis, maximum likelihood estimation, Bayes estimation, bootstrap confidence interval, 62F10, 62F12, 62F40, 62F15

## Abstract

This paper discussed the estimation of stress-strength reliability parameter R=P(Y<X) based on complete samples when the stress-strength are two independent Poisson half logistic random variables (PHLD). We have addressed the estimation of *R* in the general case and when the scale parameter is common. The classical and Bayesian estimation (BE) techniques of *R* are studied. The maximum likelihood estimator (MLE) and its asymptotic distributions are obtained; an approximate asymptotic confidence interval of *R* is computed using the asymptotic distribution. The non-parametric percentile bootstrap and student’s bootstrap confidence interval of *R* are discussed. The Bayes estimators of *R* are computed using a gamma prior and discussed under various loss functions such as the square error loss function (SEL), absolute error loss function (AEL), linear exponential error loss function (LINEX), generalized entropy error loss function (GEL) and maximum a posteriori (MAP). The Metropolis–Hastings algorithm is used to estimate the posterior distributions of the estimators of *R*. The highest posterior density (HPD) credible interval is constructed based on the SEL. Monte Carlo simulations are used to numerically analyze the performance of the MLE and Bayes estimators, the results were quite satisfactory based on their mean square error (MSE) and confidence interval. Finally, we used two real data studies to demonstrate the performance of the proposed estimation techniques in practice and to illustrate how PHLD is a good candidate in reliability studies.

## 1. Introduction

In the context of the mechanical reliability of a system or materials, it is very important to study the system performance referred to as the stress–strength parameter. Suppose a component has stress *X* and is subjected to a strength *Y*, then R=P(Y<X) defined the system performance, and its called the stress–strength parameter. The system will fail if and only if the stress applied is greater than the strength. A good design in practice is such a way that the strength is always greater than the expected stress. Since the [1] proclaim that numerical values of *R* make more sense to researchers particularly those in the medical profession and point out that *R* can be estimated under many distributional assumptions not necessarily the normality, thus giving rise to using other distributions than the normal distribution when normality is obviously inappropriate. In statistical mechanics, inference about the stress–strength parameter based on a complete or censored sample has attracted many researchers over decades and the problem of estimating *R* under different conditions has been widely studied. It is usually considered that *R* has a greater interest in reliability studies but nevertheless, *R* is an important measure in fields other than reliability since it is a measure of the difference between two populations. For example, *R* is called a measure of the treatment effect in a case where *Y* is the response of a control group and *X* represents a treatment group. When *Y* is the strength of a rocket chamber and *X* is maximal chamber pressure which is generated when a solid propellant is ignited *R* is the probability that the engine will be fired successfully [2]. *R* has an application used in comparing the strength of two types of steel [3], more applications of the stress–strength in engineering, biomedical sciences, and finance can be found in ([4] Chap. 7).

Many authors have studied the statistical inference of stress-strength model *R* in different viewpoints. For instance, for independent random variables *X* and *Y* that follow: half logistic distribution [5], Burr type X distribution [6], normal distribution [7,8], skew normal random distribution [9,10], generalized gamma distribution [11,12], logistic distribution [13], generalized logistic distribution [14], Laplace distribution [15], generalized exponential distribution [16], two-parameter exponential distribution [17,18], exponential random variables with the common location parameter [19], generalized quadratic hazard rate distribution [20], Fréchet distribution [21], power Lindley distribution [22], quadratic hazard rate-geometric distribution [23], generalized exponential Poisson [24], beta distribution [25], beta-Erlang truncated exponential [26], exponentiated half logistic-Poisson [27], Pareto distribution [28], Weibull distribution [29,30,31] among others.

In other viewpoints, ref. [32] estimated *R* for the Weibull based on the hybrid censored data, ref. [33] consider the estimation of *R* for the Weibull random variable case on progressively type-II censored data, ref. [34] discussed the estimation of *R* for the generalized exponential based on data on records, ref. [35] investigate the estimation of *R* based on Burr type XII distribution under hybrid progressive censored samples, ref. [36] estimate *R* based on independent Lomax under type-II hybrid censored samples. ref. [37] introduced a new flexible two-parameter lifetime model called Poisson half logistic distribution (PHLD). The cumulative distribution (F(x)) and the density function (f(x)) of a random variable X>0 having the PHLD with parameter α,λ>0 are given by

$$F\left(x\right)=\frac{e^{\lambda \Delta (x)}-1}{e^{\lambda }-1},$$

where, $\Delta \left(x\right)=\frac{1-e^{-\alpha x}}{1+e^{-\alpha x}}$, and

(1) $$f\left(x\right)=\frac{2\alpha \lambda e^{-\alpha x+\lambda \Delta (x)}}{\left(e^{\lambda }-1\right){\left[1+e^{-\alpha x}\right]}^{2}},$$

respectively. The quantile function of PHLD can be used for random number generation by random sampling from the uniform (0,1) distribution.

**Proposition** **1** ([37])**.** *The p^th^ quantile of the PHLD is given by*

(2) $$x_{p}=\frac{-1}{\alpha }\log\left[\frac{1-\xi }{1+\xi }\right],\;\;0<p<1,$$

*where $\xi =\frac{1}{\lambda }\log\left[p\left(e^{\lambda }-1\right)+1\right]$.*

**Proposition** **2** ([37])**.** *If the random variable X has PHLD with pdf (1), then the r^th^ moment of X is given, for r=1,2,3,…, by*

$$\mu_{r}=E\left[X^{r}\right]=\sum_{l=0}^{N}\;\varpi_{\ell }\frac{2}{{\left(1-\hbar_{\ell }\right)}^{2}}{\left(\frac{1+\hbar_{\ell }}{1-\hbar_{\ell }}\right)}^{r}f\left(\frac{1+\hbar_{\ell }}{1-\hbar_{\ell }}\right),$$

*where the $\varpi_{\ell }$ are the zeros and the corresponding Christoffel numbers of the Legendre–Gauss quadrature formula on the interval (−1,1), see [38].*

The *r*-th moment of *X* can also be represented in similar way to [39] by using the following series expansion in (3). For a real and non-integer *n* and |z|<a, we have

(3) (a+z)−n=∑i=0∞−nia−n−izi.

After some algebra the r*^th^* moment become:

E[Xr]=∑i=0∞∑w=0∞−i−2w2(−1)rλi+1(eλ−1)αri!B0r(i+1,w+1),

where $B_{kd}\left(a,b\right)=\frac{\partial^{k+d}B\left(a,b\right)}{\partial a^{k}\partial b^{d}}$ is the partial derivative of a beta function $B_{kd}\left(a,b\right)$.

The PHLD has been receiving much attention in recent years as a model for various applications, one can see the performance of PHLD in an application to the remission times (in months) of a random sample of 128 bladder cancer patients [40], and in the analysis of failure times of ball bearings in million revolutions [37].

In this paper, we are aiming at the estimation of the stress–strength parameter *R* from independent random variables with a PHLD distribution, the classical maximum likelihood method and Bayesian estimation techniques were discussed and analyzed numerically by simulation studies. Two sets of real data analysis are provided for illustration.

The rest of the paper follows: In Section 2, we provide the estimation of *R* in the general case and its maximum likelihood estimation, asymptotic distribution, and confidence interval, the bootstrap confidence interval is also considered. In Section 3 the estimation of *R* with one common parameter is studied also the maximum likelihood estimation (MLE), asymptotic distribution, confidence interval (CI), and bootstrap confidence interval are analyzed. In Section 4, Bayes estimation of *R* both in the general case and in the case of common scale parameter is proposed. The estimation of *R* based on various loss functions such as the square error loss function (SEL), absolute error loss function (AEL), maximum a posteriori (MAP), linear exponential loss function (LINEX), and general entropy loss function (GEL) are discussed. Further, the highest posterior density (HPD) credible interval for *R* is constructed. In Section 5, the estimation techniques were analyzed numerically by simulation studies. Two real data studies consisting of four different data set were given for illustration in Section 6. In Section 7, we provide a conclusion.

## 2. Estimation of *R* in General Case

In this section, we derive the expression of *R* in the general case and parameter estimation by maximum likelihood estimation. The asymptotic confidence interval and the bootstrap confidence interval of *R* are discussed.

Let X∼ PHLD1($\alpha_{1},\lambda_{1}$) and Y∼ PHLD2($\alpha_{2},\lambda_{2}$), let $f_{1}\left(x\right)$ be the density of *X* and $F_{2}\left(y\right)$ be the cumulative distribution of *Y* given by

$$f_{1}\left(x\right)=\frac{2\alpha_{1}\lambda_{1}e^{-\alpha_{1}x+\lambda_{1}\Delta_{1}\left(x\right)}}{\left(e^{\lambda_{1}}-1\right){\left[1+e^{-\alpha_{1}x}\right]}^{2}},\;x,\alpha_{1},\lambda_{1}>0,$$

where $\Delta_{1}\left(x\right)=\frac{1-e^{-\alpha_{1}x}}{1+e^{-\alpha_{1}x}}$, and

$$F_{2}\left(y\right)=\frac{e^{\lambda_{2}\Delta_{2}\left(y\right)}-1}{e^{\lambda_{2}}-1},\;y,\alpha_{2},\lambda_{2}>0,$$

where $\Delta_{2}\left(y\right)=\frac{1-e^{-\alpha_{2}y}}{1+e^{-\alpha_{2}y}}$, then, the strength-stress parameter *R* is derive as

$$\begin{array}{cc} R & =\int_{0}^{\infty }f_{1}\left(x\right)F_{2}\left(x\right)dx=\frac{2\alpha_{1}\lambda_{1}}{\left(e^{\lambda_{1}}-1\right)\left(e^{\lambda_{2}}-1\right)}\int_{0}^{\infty }\frac{e^{-\alpha_{1}x+\lambda_{1}\Delta_{1}\left(x\right)}\left(e^{\lambda_{2}\Delta_{2}\left(x\right)}-1\right)}{{\left[1+e^{-\alpha_{1}x}\right]}^{2}}dx\\ \; & =\frac{2\alpha_{1}\lambda_{1}}{\left(e^{\lambda_{1}}-1\right)\left(e^{\lambda_{2}}-1\right)}\int_{0}^{\infty }\frac{e^{-\alpha_{1}x+\lambda_{1}\Delta_{1}\left(x\right)}e^{\lambda_{2}\Delta_{2}\left(x\right)}}{{\left[1+e^{-\alpha_{1}x}\right]}^{2}}dx-\frac{1}{e^{\lambda_{2}}-1}. \end{array}$$

Let $u=e^{-x}$, then $u^{\alpha_{1}}=e^{-\alpha_{1}x}$, and $u^{\alpha_{2}}=e^{-\alpha_{2}x}$, thus,

(4) $$R=\frac{2\alpha_{1}\lambda_{1}}{\left(e^{\lambda_{1}}-1\right)\left(e^{\lambda_{2}}-1\right)}\int_{0}^{1}\frac{u^{\alpha_{1}-1}}{{\left(1+u^{\alpha_{1}}\right)}^{2}}\;e^{\lambda_{1}\left(\frac{1-u^{\alpha_{1}}}{1+u^{\alpha_{1}}}\right)}\;e^{\lambda_{2}\left(\frac{1-u^{\alpha_{2}}}{1+u^{\alpha_{2}}}\right)}du-\frac{1}{e^{\lambda_{2}}-1}.$$

The above integral can be computed numerically, but we can represent R in a series form by solvinging the integral part as follows. Let defined *B* as

$$B=\frac{2\alpha_{1}\lambda_{1}}{\left(e^{\lambda_{1}}-1\right)\left(e^{\lambda_{2}}-1\right)}\int_{0}^{1}\frac{u^{\alpha_{1}-1}}{{\left(1+u^{\alpha_{1}}\right)}^{2}}\;e^{\lambda_{1}\left(\frac{1-u^{\alpha_{1}}}{1+u^{\alpha_{1}}}\right)}\;e^{\lambda_{2}\left(\frac{1-u^{\alpha_{2}}}{1+u^{\alpha_{2}}}\right)}du.$$

Let $v=1-u^{\alpha_{1}}$, this implies $du=-dv/(\alpha_{1}u^{\alpha_{1}-1})$, and $u^{\alpha_{2}}={\left(1-v\right)}^{\frac{\alpha_{2}}{\alpha_{1}}}$, therefore,

$$B=\frac{2\lambda_{1}}{\left(e^{\lambda_{1}}-1\right)\left(e^{\lambda_{2}}-1\right)}\int_{0}^{1}\frac{1}{{\left(2-v\right)}^{2}}\;e^{\lambda_{1}\left(\frac{v}{2-v}\right)}\;e^{\lambda_{2}\left(\frac{1-{\left(1-v\right)}^{\frac{\alpha_{2}}{\alpha_{1}}}}{1+{\left(1-v\right)}^{\frac{\alpha_{2}}{\alpha_{1}}}}\right)}dv.$$

Recall that for |z|<1,

(5) (1−z)−s=∑k=0∞s+k−1kzk,

and by the exponential expansion we get

$$B=\frac{2\lambda_{1}}{\left(e^{\lambda_{1}}-1\right)\left(e^{\lambda_{2}}-1\right)}\sum_{i,j=0}^{\infty }\frac{\lambda_{1}^{i}\lambda_{2}^{j}}{i!\;j!}\int_{0}^{1}\frac{v^{i}}{{\left(2-v\right)}^{2+i}}\;{\left(\frac{1-{\left(1-v\right)}^{\frac{\alpha_{2}}{\alpha_{1}}}}{1+{\left(1-v\right)}^{\frac{\alpha_{2}}{\alpha_{1}}}}\right)}^{j}dv,$$

by the expansion in (5) and the generalized binomial expansion we obtain

B=2λ1(eλ1−1)(eλ2−1)∑i,j=0∞∑k,r=0∞∑l=0ji+k+1kj+r−1rjl(−1)k+l+rλ1iλ2ji!j!∫01vi(1−v)α2α1(r+l)+kdv,

thus,

(6) $$B=\sum_{i,j=0}^{\infty }\sum_{k,r=0}^{\infty }C_{i,j,k,l,r}\;B\left(i+1,\;\frac{\alpha_{2}}{\alpha_{1}}\left(r+l\right)+k+1\right),$$

where, Ci,j,k,l,r=∑l=0ji+k+1kj+r−1rjl2(−1)k+l+rλ1i+1λ2ji!j!(eλ1−1)(eλ2−1).

Hence, by putting (6) in (4), *R* can be approximated as

(7) $$R=\sum_{i,j=0}^{\infty }\sum_{k,r=0}^{\infty }C_{i,j,k,l,r}\;B\left(i+1,\;\frac{\alpha_{2}}{\alpha_{1}}\left(r+l\right)+k+1\right)-\frac{1}{e^{\lambda_{2}}-1}.$$

### 2.1. Maximum Likelihood Estimation

Suppose $x_{1},x_{2},\ldots ,x_{n_{1}}$ is a random sample of size $n_{1}$ from PHLD1$\left(\alpha_{1},\lambda_{1}\right)$ and $y_{1},y_{2},\ldots ,y_{n_{2}}$ is an independent random sample of size $n_{2}$ from PHLD2$\left(\alpha_{2},\lambda_{2}\right)$. The log likelihood function $L\left(\alpha_{1},\alpha_{2},\lambda_{1},\lambda_{2}\right)=L\left(\theta \right)$ is given by (8) below, where θ is a vector of parameters given by $\theta ={\left(\alpha_{1},\alpha_{2},\lambda_{1},\lambda_{2}\right)}^{T}$.

(8) $$\begin{array}{cc} \log L & =\sum_{i=1}^{n_{1}}\log f_{X}\left(x_{i}\right)+\sum_{j=1}^{n_{2}}\log f_{Y}\left(y_{j}\right)\\ \; & =\left(n_{1}+n_{2}\right)\log2+n_{1}\log\alpha_{1}+n_{2}\alpha_{2}+n_{1}\log\lambda_{1}+n_{2}\log\lambda_{2}-n_{1}\log\left(e^{\lambda_{1}}-1\right)\\ \; & -n_{2}\log\left(e^{\lambda_{2}}-1\right)-\alpha_{1}\sum_{i=1}^{n_{1}}x_{i}-\alpha_{2}\sum_{j=1}^{n_{2}}y_{j}-2\sum_{i=1}^{n_{1}}\log\left(1+e^{-\alpha_{1}x_{i}}\right)\\ \; & -2\sum_{j=1}^{n_{2}}\log\left(1+e^{-\alpha_{2}y_{j}}\right)+\lambda_{1}\sum_{i=1}^{n_{1}}\Delta_{1}\left(x_{i}\right)+\lambda_{2}\sum_{j=1}^{n_{2}}\Delta_{2}\left(y_{j}\right). \end{array}$$

To obtain the estimators of θ that is $\hat{\theta }={\left({\hat{\alpha }}_{1},{\hat{\alpha }}_{2},{\hat{\lambda }}_{1},{\hat{\lambda }}_{2}\right)}^{T}$, we need to solve the following nonlinear Equations (9)–(12) below. These equations cannot be solved analytically, but by the use of numerical optimizations available in Mathematica, Matlab or R.

(9) $$\begin{array}{cc} \frac{\partial L}{\partial \alpha_{1}} & =\frac{n_{1}}{\alpha_{1}}-\sum_{i=1}^{n_{1}}x_{i}+2\sum_{i=1}^{n_{1}}\frac{x_{i}\;e^{-\alpha_{1}x_{i}}}{1+e^{-\alpha_{1}x_{i}}}+2\lambda_{1}\sum_{i=1}^{n_{1}}\frac{x_{i}\;e^{-\alpha_{1}x_{i}}}{{\left(1+e^{-\alpha_{1}x_{i}}\right)}^{2}}, \end{array}$$

(10) $$\begin{array}{cc} \frac{\partial L}{\partial \alpha_{2}} & =\frac{n_{2}}{\alpha_{2}}-\sum_{j=1}^{n_{2}}y_{j}+2\sum_{j=1}^{n_{2}}\frac{y_{j}\;e^{-\alpha_{2}y_{j}}}{1+e^{-\alpha_{2}y_{j}}}+2\lambda_{2}\sum_{j=1}^{n_{2}}\frac{y_{j}\;e^{-\alpha_{2}y_{j}}}{{\left(1+e^{-\alpha_{2}y_{j}}\right)}^{2}}, \end{array}$$

(11) $$\begin{array}{cc} \frac{\partial L}{\partial \lambda_{1}} & =\frac{n_{1}}{\lambda_{1}}-\frac{n_{1}\;e^{\lambda_{1}}}{e^{\lambda_{1}}-1}+\sum_{i=1}^{n_{1}}\Delta_{1}\left(x_{i}\right), \end{array}$$

(12) $$\begin{array}{cc} \frac{\partial L}{\partial \lambda_{2}} & =\frac{n_{2}}{\lambda_{2}}-\frac{n_{2}\;e^{\lambda_{2}}}{e^{\lambda_{2}}-1}+\sum_{j=1}^{n_{2}}\Delta_{2}\left(y_{j}\right). \end{array}$$

Once $\hat{\theta }$ is computed, we can get the maximum likelihood estimator of R(θ) say $\hat{R}\left(\hat{\theta }\right)$ from (7) or (4).

### 2.2. Asymptotic Distribution and Confidence Interval

In this subsection, we derived the asymptotic distribution of $\hat{\theta }={\left({\hat{\alpha }}_{1},{\hat{\alpha }}_{2},{\hat{\lambda }}_{1},{\hat{\lambda }}_{2}\right)}^{T}$ then we derive the asymptotic distribution of $\hat{R}$, and the asymptotic confidence intervals of *R*. We first require the Fisher information matrix defined by I(θ)=−E[J(θ)], where $J\left(\theta \right)=\frac{\partial^{2}L}{\partial \theta \partial \theta^{T}}$, thus,

$$I\left(\theta \right)=-\left(\begin{array}{cccc} I_{\alpha_{1}\alpha_{1}} & I_{\alpha_{1}\alpha_{2}} & I_{\alpha_{1}\lambda_{1}} & I_{\alpha_{1}\lambda_{2}}\\ I_{\alpha_{2}\alpha_{1}} & I_{\alpha_{2}\alpha_{2}} & I_{\alpha_{2}\lambda_{1}} & I_{\alpha_{2}\lambda_{2}}\\ I_{\lambda_{1}\alpha_{1}} & I_{\lambda_{1}\alpha_{2}} & I_{\lambda_{1}\lambda_{1}} & I_{\lambda_{1}\lambda_{2}}\\ I_{\lambda_{2}\alpha_{1}} & I_{\lambda_{2}\alpha_{2}} & I_{\lambda_{2}\lambda_{1}} & I_{\lambda_{2}\lambda_{2}} \end{array}\right).$$

The elements of J(θ) and the computation of the element of *I* are provided in Appendix A. Before we provided the elements of I(θ) we need the following Lemma 1 used to compute the elements of I(θ).

**Lemma** **1.** *Let $x,\;\alpha ,\;\gamma_{4}>0$, $\gamma_{1},\gamma_{2},\gamma_{3}\in N$, let*

$$\nabla \left(\alpha ,\gamma_{1},\gamma_{2},\gamma_{3},\gamma_{4}\right)=\int_{0}^{\infty }\frac{x^{\gamma_{1}}\;e^{-\gamma_{2}\alpha x+\gamma_{4}\Delta \left(x\right)}}{{\left[1+e^{-\alpha x}\right]}^{\gamma_{3}}}dx,$$

*then,*

$$\nabla \left(\alpha ,\gamma_{1},\gamma_{2},\gamma_{3},\gamma_{4}\right)=\sum_{m,s=0}^{\infty }\phi_{m,s}\;B_{0,\gamma_{1}}\left(m+1,\;\gamma_{2}+s\right),$$

*where ϕm,s=γ3+m+s−1sγ4m(−1)γ1+sm!αγ1+1 and $B_{t,\gamma_{1}}\left(a,b\right)=\frac{\partial^{t+\gamma_{1}}}{\partial a^{t}\;\partial b^{\gamma_{1}}}\;B\left(a,\;b\right)$. In particular, when $\gamma_{1}=1,\;2,$ we have*

$$\nabla \left(\alpha ,1,\gamma_{2},\gamma_{3},\gamma_{4}\right)=\sum_{m,s=0}^{\infty }\phi_{m,s}\;\left(\psi^{0}\left(\gamma_{2}+s\right)-\psi^{0}\left(m+\gamma_{2}+s+1\right)\right)B\left(m+1,\;\gamma_{2}+s\right),$$

*and*

$$\begin{array}{cc} \left.\nabla \left(\alpha ,2,\gamma_{2},\gamma_{3},\gamma_{4}\right)=\sum_{m,s=0}^{\infty }\phi_{m,s}\;\right. & \left.({\left[\psi^{0}\left(\gamma_{2}+s\right)-\psi^{0}\left(m+\gamma_{2}+s+1\right)\right]}^{2}\right.\\ \; & \left.+\psi^{1}\left(\gamma_{2}+s\right)-\psi^{1}\left(m+\gamma_{2}+s+1\right))B\left(m+1,\;\gamma_{2}+s\right),\right. \end{array}$$

*where $\psi^{m}\left(z\right)=\frac{d^{m}}{dz^{m}}\psi \left(z\right)=\frac{d^{m+1}}{dz^{m+1}}\ln\Gamma \left(z\right)$ is called polygamma function, and $\psi \left(z\right)=\psi^{0}\left(z\right)=\frac{d}{dz}\ln\Gamma \left(z\right)=\frac{\Gamma^{\prime }\left(z\right)}{\Gamma (z)}$ is called the digamma function.*

**Proof.**  Let

$$\nabla =\int_{0}^{\infty }\frac{x^{\gamma_{1}}\;e^{-\gamma_{2}\alpha x+\gamma_{4}\Delta \left(x\right)}}{{\left[1+e^{-\alpha x}\right]}^{\gamma_{3}}}dx,$$

by the exponential expansion we have

$$\nabla =\sum_{m=0}^{\infty }\frac{\gamma_{4}^{m}}{m!}\int_{0}^{\infty }\frac{x^{\gamma_{1}}\;{\left(1-e^{-\alpha x}\right)}^{m}\;e^{-\gamma_{2}\alpha x}}{{\left(1+e^{-\alpha x}\right)}^{\gamma_{3}+m}}\;dx.$$

Let $u=e^{-\alpha x}$, then, $e^{-\gamma_{2}\alpha x}={\left(1-u\right)}^{\gamma_{2}}$, in similar way to the computation of *R* we get

∇=∑m=0∞γ4m(−1)γ1m!αγ1+1∫01logγ1(1−u)um(1−u)γ2−1(1+(1−u))γ3+mdu,=∑m,s=0∞γ3+m+s−1sγ4m(−1)γ1+sm!αγ1+1∫01logγ1(1−u)um(1−u)γ2+s−1du,=∑m,s=0∞ϕm,sB0,γ1(m+1,γ2+s),

where ϕm,s=γ3+m+s−1sγ4m(−1)γ1+sm!αγ1+1 and $B_{t,\gamma_{1}}\left(a,b\right)=\frac{\partial^{t+\gamma_{1}}}{\partial a^{t}\;\partial b^{\gamma_{1}}}\;B\left(a,\;b\right)$. □

Hence, the elements of I(θ) are derived using the Lemma 1 as:

$$\begin{array}{cc} I_{\alpha_{1}\alpha_{1}} & =-\frac{n_{1}}{\alpha_{1}^{2}}-\frac{4n_{1}\alpha_{1}\lambda_{1}}{\left(e^{\lambda_{1}}-1\right)}\nabla \left(\alpha_{1},2,2,3,\lambda_{1}\right)+\frac{4n_{1}\alpha_{1}\lambda_{1}}{\left(e^{\lambda_{1}}-1\right)}\nabla \left(\alpha_{1},2,3,4,\lambda_{1}\right),\\ \; & -\frac{4n_{1}\alpha_{1}\lambda_{1}^{2}}{\left(e^{\lambda_{1}}-1\right)}\nabla \left(\alpha_{1},2,2,4,\lambda_{1}\right)+\frac{8n_{1}\alpha_{1}\lambda_{1}^{2}}{\left(e^{\lambda_{1}}-1\right)}\nabla \left(\alpha_{1},2,3,5,\lambda_{1}\right), \end{array}$$

$$\begin{array}{cc} I_{\alpha_{2}\alpha_{2}} & =-\frac{n_{2}}{\alpha_{2}^{2}}-\frac{4n_{2}\alpha_{2}\lambda_{2}}{\left(e^{\lambda_{2}}-1\right)}\nabla \left(\alpha_{2},2,2,3,\lambda_{2}\right)+\frac{4n_{2}\alpha_{2}\lambda_{2}}{\left(e^{\lambda_{2}}-1\right)}\nabla \left(\alpha_{2},2,3,4,\lambda_{2}\right),\\ \; & -\frac{4n_{2}\alpha_{2}\lambda_{2}^{2}}{\left(e^{\lambda_{2}}-1\right)}\nabla \left(\alpha_{2},2,2,4,\lambda_{2}\right)+\frac{8n_{2}\alpha_{2}\lambda_{2}^{2}}{\left(e^{\lambda_{2}}-1\right)}\nabla \left(\alpha_{2},2,3,5,\lambda_{2}\right),\\ I_{\lambda_{1}\lambda_{1}} & =-\frac{n_{1}}{\lambda_{1}^{2}}-\frac{n_{1}\;e^{\lambda_{1}}}{{\left(e^{\lambda_{1}}-1\right)}^{2}},\\ I_{\lambda_{2}\lambda_{2}} & =-\frac{n_{2}}{\lambda_{2}^{2}}-\frac{n_{2}\;e^{\lambda_{2}}}{{\left(e^{\lambda_{2}}-1\right)}^{2}},\\ I_{\alpha_{1}\lambda_{1}} & =\frac{4n_{1}\lambda_{1}\alpha_{1}}{e^{\lambda_{1}}-1}\nabla \left(\alpha_{1},1,2,4,\lambda_{1}\right),\\ I_{\alpha_{2}\lambda_{2}} & =\frac{4n_{2}\lambda_{2}\alpha_{2}}{e^{\lambda_{2}}-1}\nabla \left(\alpha_{2},1,2,4,\lambda_{2}\right),\\ I_{\alpha_{2}\alpha_{1}} & =I_{\alpha_{1}\alpha_{2}}=I_{\alpha_{1}\lambda_{2}}=I_{\lambda_{2}\alpha_{1}}=I_{\alpha_{2}\lambda_{1}}=I_{\lambda_{1}\alpha_{2}}=I_{\lambda_{1}\lambda_{2}}=I_{\lambda_{2}\lambda_{1}}=0. \end{array}$$

Thus, we can establish the asymptotic distribution for the MLEs.

**Lemma** **2.** *As $n_{1}\to \infty$, $n_{2}\to \infty$, then, $\sqrt{n_{1}+n_{2}}\left(\hat{\theta }-\theta \right)\to N_{4}\left(0,I^{-1}\left(\theta \right)\right)$, where*

$$\lim_{n_{1},n_{2}\to \infty }\frac{1}{n_{1}+n_{2}}I^{-1}\left(\theta \right)=\left(\begin{array}{cccc} Var(\hat{\alpha_{1}}) & Cov(\hat{\alpha_{1}}\hat{\alpha_{2}}) & Cov(\hat{\alpha_{1}}\hat{\lambda_{1}}) & Cov(\hat{\alpha_{1}}\hat{\lambda_{2}})\\ Cov(\hat{\alpha_{2}}\hat{\alpha_{1}}) & Var(\hat{\alpha_{2}}) & Cov(\hat{\alpha_{2}}\hat{\lambda_{1}}) & Cov(\hat{\alpha_{2}}\hat{\lambda_{2}})\\ Cov(\hat{\lambda_{1}}\hat{\alpha_{1}}) & Cov(\hat{\lambda_{1}}\hat{\alpha_{2}}) & Var(\hat{\lambda_{1}}) & Cov(\hat{\lambda_{1}}\hat{\lambda_{2}})\\ Cov(\hat{\lambda_{2}}\hat{\alpha_{1}}) & Cov(\hat{\lambda_{2}}\hat{\alpha_{2}}) & Cov(\hat{\lambda_{2}}\hat{\lambda_{1}}) & Var(\hat{\lambda_{2}}) \end{array}\right)$$

**Proof.**  follows from the asymptotic normality of MLE. □

To, establish the asymptotic distribution of *R*, we need to compute the partial derivative of R(θ), say $B\left(\theta \right)={\left(\frac{\partial R}{\partial \alpha_{1}},\frac{\partial R}{\partial \alpha_{2}},\frac{\partial R}{\partial \lambda_{1}},\frac{\partial R}{\partial \lambda_{2}}\right)}^{T}$ as follows, but before that, we need the following Lemma 3.

**Lemma** **3.** *Let x>0, $\Theta ={\left(\alpha_{1},\alpha_{2},\lambda_{1},\lambda_{2},\delta_{1},\delta_{2},\delta_{3},\delta_{4},\delta_{5},\delta_{6}\right)}^{T}>0$, let,*

$$\zeta \left(\Theta \right)=\int_{0}^{\infty }\frac{x^{\delta_{1}}\;{\left(1-e^{-\alpha_{1}x}\right)}^{\delta_{3}}\;{\left(1-e^{-\alpha_{2}x}\right)}^{\delta_{4}}}{{\left(1+e^{-\alpha_{1}x}\right)}^{\delta_{5}}\;{\left(1+e^{-\alpha_{2}x}\right)}^{\delta_{6}}}e^{-\delta_{2}x+\lambda_{1}\Delta_{1}\left(x\right)+\lambda_{2}\Delta_{2}\left(x\right)}\;dx,$$

*then*

$$\zeta \left(\Theta \right)=\sum_{i,j=0}^{\infty }\sum_{l,m=0}^{\infty }D_{i,j,k,l,m}\;B_{0,\delta_{1}}\left(\delta_{3}+i+1,\;\frac{\delta_{2}-1}{\alpha_{1}}+\frac{\alpha_{2}}{\alpha_{1}}\left(k+m\right)+\alpha_{1}l\right),$$

*where Di,j,k,l,m=∑k=0δ4+j(−1)δ1+k+l+mλ1iλ2ji!j!α1α1+1δ5+i+l−1lδ6+j+m−1mδ4+jk.*

**Proof.**  Let

$$\zeta \left(\Theta \right)=\int_{0}^{\infty }\frac{x^{\delta_{1}}\;{\left(1-e^{-\alpha_{1}x}\right)}^{\delta_{3}}\;{\left(1-e^{-\alpha_{2}x}\right)}^{\delta_{4}}}{{\left(1+e^{-\alpha_{1}x}\right)}^{\delta_{5}}\;{\left(1+e^{-\alpha_{2}x}\right)}^{\delta_{6}}}e^{-\delta_{2}x+\lambda_{1}\Delta_{1}\left(x\right)+\lambda_{2}\Delta_{2}\left(x\right)}\;dx,$$

by applying the exponential expansion and letting $u=e^{-x}$ in similar way to the computation of (7) we get

$$\zeta \left(\Theta \right)=\sum_{i,j=0}^{\infty }\frac{{\left(-1\right)}^{\delta_{1}}\lambda_{1}^{i}\lambda_{2}^{j}}{i!\;j!}\int_{0}^{1}\;\frac{\log^{\delta_{1}}u\;u^{\delta_{2}-1}\;{\left(1-u^{\alpha_{1}}\right)}^{\delta_{3}+i}\;{\left(1-u^{\alpha_{2}}\right)}^{\delta_{4}+j}}{{\left(1+u^{\alpha_{1}}\right)}^{\delta_{5}+i}\;{\left(1+u^{\alpha_{2}}\right)}^{\delta_{6}+j}}\;du,$$

letting $v=1-u^{\alpha_{1}}$ we have

$$\zeta \left(\Theta \right)=\sum_{i,j=0}^{\infty }\frac{{\left(-1\right)}^{\delta_{1}}\lambda_{1}^{i}\lambda_{2}^{j}}{i!\;j!\;\alpha_{1}^{\alpha_{1}+1}}\int_{0}^{1}\;\frac{\log^{\delta_{1}}\left(1-v\right)\;{\left(1-v\right)}^{\frac{\delta_{2}-1}{\alpha_{1}}-1}\;v^{\delta_{3}+i}\;{\left(1-{\left(1-v\right)}^{\frac{\alpha_{2}}{\alpha_{1}}}\right)}^{\delta_{4}+j}}{{\left(1+{\left(1-v\right)}^{\alpha_{1}}\right)}^{\delta_{5}+i}\;{\left(1+{\left(1-v\right)}^{\frac{\alpha_{2}}{\alpha_{1}}}\right)}^{\delta_{6}+j}}\;dv,$$

by the generalized binomial expansion, finally we obtain

$$\zeta \left(\Theta \right)=\sum_{i,j=0}^{\infty }\sum_{l,m=0}^{\infty }D_{i,j,k,l,m}\int_{0}^{1}\;\log^{\delta_{1}}\left(1-v\right)\;v^{\delta_{3}+i}\;{\left(1-v\right)}^{\frac{\delta_{2}-1}{\alpha_{1}}+\frac{\alpha_{2}}{\alpha_{1}}\left(k+m\right)+\alpha_{1}l-1}dv,$$

hence,

$$\zeta \left(\Theta \right)=\sum_{i,j=0}^{\infty }\sum_{l,m=0}^{\infty }D_{i,j,k,l,m}\;B_{0,\delta_{1}}\left(\delta_{3}+i+1,\;\frac{\delta_{2}-1}{\alpha_{1}}+\frac{\alpha_{2}}{\alpha_{1}}\left(k+m\right)+\alpha_{1}l\right),$$

where Di,j,k,l,m=∑k=0δ4+j(−1)δ1+k+l+mλ1iλ2ji!j!α1α1+1δ5+i+l−1lδ6+j+m−1mδ4+jk. □

Now, the derivative of R(θ) can be computed by applying the Lemma 3.

$$\begin{array}{cc} \frac{\partial R}{\partial \lambda_{1}} & =\frac{2\alpha_{1}}{\left(e^{\lambda_{1}}-1\right)\left(e^{\lambda_{2}}-1\right)}\int_{0}^{\infty }\;\frac{e^{-\alpha_{1}x+\lambda_{1}\Delta_{1}\left(x\right)+\lambda_{2}\Delta_{2}\left(x\right)}}{{\left(1+e^{-\alpha_{1}x}\right)}^{2}}dx\\ \; & -\frac{2\alpha_{1}\lambda_{1}e^{\lambda_{1}}}{\left(e^{\lambda_{1}}-1\right)\left(e^{\lambda_{2}}-1\right)}\int_{0}^{\infty }\;\frac{e^{-\alpha_{1}x+\lambda_{1}\Delta_{1}\left(x\right)+\lambda_{2}\Delta_{2}\left(x\right)}}{{\left(1+e^{-\alpha_{1}x}\right)}^{2}}dx\\ \; & +\frac{2\alpha_{1}\lambda_{1}}{\left(e^{\lambda_{1}}-1\right)\left(e^{\lambda_{2}}-1\right)}\int_{0}^{\infty }\;\frac{e^{-\alpha_{1}x+\lambda_{1}\Delta_{1}\left(x\right)+\lambda_{2}\Delta_{2}\left(x\right)}\left(1-e^{-\alpha_{1}x}\right)}{{\left(1+e^{-\alpha_{1}x}\right)}^{3}}dx\\ \; & =\frac{2\alpha_{1}}{\left(e^{\lambda_{1}}-1\right)\left(e^{\lambda_{2}}-1\right)}\zeta \left(\alpha_{1},\alpha_{2},\lambda_{1},\lambda_{2},0,\alpha_{1},0,0,2,0\right)\\ \; & -\frac{2\alpha_{1}\lambda_{1}e^{\lambda_{1}}}{{\left(e^{\alpha_{1}}-1\right)}^{2}\left(e^{\alpha_{2}}-1\right)}\zeta \left(\alpha_{1},\alpha_{2},\lambda_{1},\lambda_{2},0,\alpha_{1},0,0,2,0\right)\\ \; & +\frac{2\alpha_{1}\lambda_{1}}{\left(e^{\lambda_{1}}-1\right)\left(e^{\lambda_{2}}-1\right)}\zeta \left(\alpha_{1},\alpha_{2},\lambda_{1},\lambda_{2},0,\alpha_{1},1,0,3,0\right),\\ \frac{\partial R}{\partial \lambda_{2}} & =-\frac{2\alpha_{1}\lambda_{1}e^{\lambda_{2}}}{\left(e^{\lambda_{1}}-1\right){\left(e^{\lambda_{2}}-1\right)}^{2}}\int_{0}^{\infty }\;\frac{e^{-\alpha_{1}x+\lambda_{1}\Delta_{1}\left(x\right)+\lambda_{2}\Delta_{2}\left(x\right)}}{{\left(1+e^{-\alpha_{1}x}\right)}^{2}}dx\\ \; & +\frac{2\alpha_{1}\lambda_{1}}{\left(e^{\lambda_{1}}-1\right)\left(e^{\lambda_{2}}-1\right)}\int_{0}^{\infty }\;\frac{e^{-\alpha_{1}x+\lambda_{1}\Delta_{1}\left(x\right)+\lambda_{2}\Delta_{2}\left(x\right)}\;\left(1-e^{-\alpha_{1}x}\right)}{{\left(1+e^{-\alpha_{1}x}\right)}^{2}\;\left(1+e^{-\alpha_{2}x}\right)}dx+\frac{e^{\lambda_{2}}}{{\left(e^{\lambda_{2}}-1\right)}^{2}}\\ \; & =-\frac{2\alpha_{1}\lambda_{1}e^{\lambda_{2}}}{\left(e^{\lambda_{1}}-1\right){\left(e^{\lambda_{2}}-1\right)}^{2}}\zeta \left(\alpha_{1},\alpha_{2},\lambda_{1},\lambda_{2},0,\alpha_{1},0,0,2,0\right)\\ \; & +\frac{2\alpha_{1}\lambda_{1}}{\left(e^{\lambda_{1}}-1\right)\left(e^{\lambda_{2}}-1\right)}\zeta \left(\alpha_{1},\alpha_{2},\lambda_{1},\lambda_{2},0,\alpha_{1},1,0,2,1\right)+\frac{e^{\lambda_{2}}}{{\left(e^{\lambda_{2}}-1\right)}^{2}},\\ \frac{\partial R}{\partial \alpha_{1}} & =\frac{2\alpha_{1}\lambda_{1}}{\left(e^{\lambda_{1}}-1\right)\left(e^{\lambda_{2}}-1\right)}\int_{0}^{\infty }\;\frac{e^{-\alpha_{1}x+\lambda_{1}\Delta_{1}\left(x\right)+\lambda_{2}\Delta_{2}\left(x\right)}}{{\left(1+e^{-\alpha_{1}x}\right)}^{2}}dx\\ \; & -\frac{2\alpha_{1}\lambda_{1}}{\left(e^{\lambda_{1}}-1\right)\left(e^{\lambda_{2}}-1\right)}\int_{0}^{\infty }\;\frac{x\;e^{-\alpha_{1}x+\lambda_{1}\Delta_{1}\left(x\right)+\lambda_{2}\Delta_{2}\left(x\right)}}{{\left(1+e^{-\alpha_{1}x}\right)}^{2}}dx\\ \; & +\frac{4\alpha_{1}\lambda_{1}}{\left(e^{\lambda_{1}}-1\right)\left(e^{\lambda_{2}}-1\right)}\int_{0}^{\infty }\;\frac{x\;e^{-2\alpha_{1}x+\lambda_{1}\Delta_{1}\left(x\right)+\lambda_{2}\Delta_{2}\left(x\right)}}{{\left(1+e^{-\alpha_{1}x}\right)}^{3}}dx\\ \; & +\frac{4\alpha_{1}\lambda_{1}^{2}}{\left(e^{\lambda_{1}}-1\right)\left(e^{\lambda_{2}}-1\right)}\int_{0}^{\infty }\;\frac{x\;e^{-2\alpha_{1}x+\lambda_{1}\Delta_{1}\left(x\right)+\lambda_{2}\Delta_{2}\left(x\right)}}{{\left(1+e^{-\alpha_{1}x}\right)}^{4}}dx \end{array}$$

$$\begin{array}{cc} \; & =\frac{2\alpha_{1}\lambda_{1}}{\left(e^{\lambda_{1}}-1\right)\left(e^{\lambda_{2}}-1\right)}\zeta \left(\alpha_{1},\alpha_{2},\lambda_{1},\lambda_{2},0,\alpha_{1},0,0,2,0\right)\\ \; & -\frac{2\alpha_{1}\lambda_{1}}{\left(e^{\lambda_{1}}-1\right)\left(e^{\lambda_{2}}-1\right)}\zeta \left(\alpha_{1},\alpha_{2},\lambda_{1},\lambda_{2},1,\alpha_{1},0,0,2,0\right)\\ \; & +\frac{4\alpha_{1}\lambda_{1}}{\left(e^{\lambda_{1}}-1\right)\left(e^{\lambda_{2}}-1\right)}\zeta \left(\alpha_{1},\alpha_{2},\lambda_{1},\lambda_{2},1,2\alpha_{1},0,0,3,0\right)\\ \; & +\frac{4\alpha_{1}\lambda_{1}^{2}}{\left(e^{\lambda_{1}}-1\right)\left(e^{\lambda_{2}}-1\right)}\zeta \left(\alpha_{1},\alpha_{2},\lambda_{1},\lambda_{2},1,2\alpha_{1},0,0,4,0\right)\\ \frac{\partial R}{\partial \alpha_{2}} & =\frac{4\alpha_{1}\lambda_{1}\lambda_{2}}{\left(e^{\lambda_{1}}-1\right)\left(e^{\lambda_{2}}-1\right)}\int_{0}^{\infty }\;\frac{x\;e^{-\left(\alpha_{1}+\alpha_{2}\right)x+\lambda_{1}\Delta_{1}\left(x\right)+\lambda_{2}\Delta_{2}\left(x\right)}}{{\left(1+e^{-\alpha_{1}x}\right)}^{2}\;{\left(1+e^{-\alpha_{2}x}\right)}^{2}}dx\\ \; & =\frac{4\alpha_{1}\lambda_{1}\lambda_{2}}{\left(e^{\lambda_{1}}-1\right)\left(e^{\lambda_{2}}-1\right)}\zeta \left(\alpha_{1},\alpha_{2},\lambda_{1},\lambda_{2},1,\alpha_{1}+\alpha_{2},0,0,2,2\right). \end{array}$$

Therefore, using Lemma 4, we obtain the asymptotic distribution of $\hat{R(\theta )}$ as

(13) $$\sqrt{n_{1}+n_{2}}\left(\hat{R}-R\right)\to N_{4}\left(0,B^{T}\left(\theta \right)I^{-1}\left(\theta \right)B\left(\theta \right)\right).$$

Thus, the asymptotic variance of $\hat{R}$ from (13) is

$$\begin{array}{cc} Var(\hat{R}) & =\frac{1}{n_{1}+n_{2}}B^{T}\left(\theta \right)I^{-1}\left(\theta \right)B\left(\theta \right)={\left(\frac{\partial R}{\partial \alpha_{1}}\right)}^{2}Var\left(\hat{\alpha_{1}}\right)+{\left(\frac{\partial R}{\partial \alpha_{2}}\right)}^{2}Var\left(\hat{\alpha_{2}}\right)\\ \; & +{\left(\frac{\partial R}{\partial \lambda_{1}}\right)}^{2}Var\left(\hat{\lambda_{1}}\right)+\left(\frac{\partial R}{\partial \lambda_{2}}\right)Var\left(\lambda_{2}\right)+2\frac{\partial R}{\partial \alpha_{1}}\frac{\partial R}{\partial \alpha_{2}}Cov\left(\hat{\alpha_{1}}\hat{\alpha_{2}}\right)\\ \; & +2\frac{\partial R}{\partial \alpha_{1}}\frac{\partial R}{\partial \lambda_{1}}Cov\left(\hat{\alpha_{1}}\hat{\lambda_{1}}\right)+2\frac{\partial R}{\partial \alpha_{1}}\frac{\partial R}{\partial \lambda_{2}}Cov\left(\hat{\alpha_{1}}\hat{\lambda_{2}}\right)\\ \; & +2\frac{\partial R}{\partial \alpha_{2}}\frac{\partial R}{\partial \lambda_{1}}Cov\left(\hat{\alpha_{2}}\hat{\lambda_{1}}\right)+2\frac{\partial R}{\partial \alpha_{2}}\frac{\partial R}{\partial \lambda_{2}}Cov\left(\hat{\alpha_{2}}\hat{\lambda_{2}}\right)\\ \; & +2\frac{\partial R}{\partial \lambda_{1}}\frac{\partial R}{\partial \lambda_{2}}Cov\left(\hat{\lambda_{1}}\hat{\lambda_{2}}\right). \end{array}$$

The asymptotic 100(1−ϵ) confidence interval $AS_{cl}$ for *R* can be constructed as

$$\hat{R}\pm Z_{\frac{\epsilon }{2}}\sqrt{Var(\hat{R})},$$

where $Z_{\frac{\epsilon }{2}}$ is the upper $\frac{\epsilon }{2}$ quantile of the standard normal distribution. Next, we consider the use of a bootstrap confidence interval preferably not for a larger sample size. The bootstrap confidence interval for a large sample may require sufficient time computationally.

### 2.3. Bootstrap Confidence Intervals for R

In this subsection, we proposed two non-parametric confidence intervals, the percentile bootstrap confidence interval ($B_{p}$), and the student’s bootstrap confidence interval ($B_{t}$) based on [41]. The procedures for the estimation of the two bootstrap confidence intervals of R are as follows.

1. Generate independent samples $x_{1},\;x_{2},\;x_{3},\;\ldots ,\;x_{n_{1}}$ from PGHLD1$\left(\alpha_{1},\lambda_{1}\right)$, and $y_{1},\;y_{2},\;y_{3},\;\ldots ,\;y_{n_{2}}$ from PGHLD2$\left(\alpha_{2},\lambda_{2}\right)$. The samples can be generated from (2) by sampling *p* from uniform distribution i.e., p∼U(0,1).
2. Generate an independent bootstrap sample $x_{1}^{*},\;x_{2}^{*},\;x_{3}^{*},\;\ldots ,\;x_{n_{1}}^{*}$ and $y_{1}^{*},\;y_{2}^{*},\;y_{3}^{*},\;\ldots ,\;y_{n_{2}}^{*}$ taken with replacement from the given samples above in the first step. Based on the bootstrap sample compute the maximum likelihood estimates of $\theta ={\left(\alpha_{1},\alpha_{2},\lambda_{1},\lambda_{2}\right)}^{T}$ say ${\hat{\theta }}^{*}={\left({\hat{\alpha }}_{1}^{*},{\hat{\alpha }}_{2}^{*},{\hat{\lambda }}_{1}^{*},{\hat{\lambda }}_{2}^{*}\right)}^{T}$ as well as the MLE of $\hat{R^{*}}\left(\hat{\theta^{*}}\right)$.
3. Repeat step 2 to 3 B-times to obtain a set of bootstrap samples of *R* say ${\hat{R}}_{j}^{*}$, j=1,2,…,B.

From the above bootstrap sample of ${\hat{R}}_{j}^{*}$ we can determine the two different bootstrap confidence intervals of *R* as follows by rearranging the sample in the order ${\hat{R}}_{1}^{*}<{\hat{R}}_{2}^{*}<{\hat{R}}_{3}^{*}<\ldots <{\hat{R}}_{B}^{*}$.

#### 2.3.1. Percentile Bootstrap Confidence Interval $\left(B_{p}\right)$:

Let ${\hat{R}}_{\left(\tau \right)}^{*}$ be the τ percentile of ${\hat{R}}_{j}^{*}$, j=1,2,3,…,B, such that

$$\frac{1}{B}\sum_{j=1}^{B}I_{{\hat{R}}_{j}^{*}\leq {\hat{R}}_{\tau }^{*}}=\tau ,\;0<\tau <1,$$

where $I_{\left(.\right)}$ is the indicator function. A 100(1−ϵ)%$B_{p}$ confidence interval of *R* is given as

$$\left({\hat{R}}_{\left(\frac{\epsilon }{2}\right)}^{*},{\hat{R}}_{\left(1-\frac{\epsilon }{2}\right)}^{*}\right).$$

#### 2.3.2. Student’s t Bootstrap Confidence Interval ($B_{t}$):

Let ${\bar{\hat{R}}}^{*}$ and $se({\hat{R}}^{*})$ be the sample mean and sample standard deviation of the ${\hat{R}}_{j}^{*}$, j=1,2,…,3, respectively, that is

$${\bar{\hat{R}}}^{*}=\frac{\sum_{j=1}^{B}{\hat{R}}_{j}^{*}}{B}\;\;and\;\;se\left({\hat{R}}^{*}\right)=\sqrt{\frac{1}{B}\sum_{j=1}^{B}{\left({\hat{R}}_{j}^{*}-{\bar{\hat{R}}}^{*}\right)}^{2}}.$$

Then, let ${\hat{t}}_{\tau }^{*}$ be the τ percentile of $\frac{{\hat{R}}_{j}^{*}-\hat{R}}{se({\hat{R}}^{*})}$, j=1,2,…,3, that is ${\hat{t}}_{\tau }^{*}$ is such that

$$\frac{1}{B}\sum_{j=1}^{B}I_{\frac{{\hat{R}}_{j}^{*}-\hat{R}}{se({\hat{R}}^{*})}\leq {\hat{t}}^{*}}=\tau ,\;0<\tau <1.$$

A 100(1−ϵ)%$B_{t}$ confidence interval of *R* is given as

$$\hat{R}\pm {\hat{t}}_{\left(\frac{\epsilon }{2}\right)}^{*}se\left({\hat{R}}^{*}\right).$$

## 3. Estimation of *R* with Common Scale Parameter α

In this section, we derived the approximation of *R* when the random variables have the same scale parameter α. The maximum likelihood estimation of *R*, the asymptotic distribution of the MLEs and *R*, and the bootstrap confidence interval of *R* are presented.

Let X∼PHLD1($\alpha ,\lambda_{1}$) and Y∼PHLD2($\alpha ,\lambda_{2}$), let $f_{1}\left(x\right)$ be the density of *X* and $F_{2}\left(y\right)$ be the cumulative distribution of *Y*, then, in similar way, the reliability *R* in this case is given by

$$\begin{array}{cc} R & =\frac{2\alpha \lambda_{1}}{\left(e^{\lambda_{1}}-1\right)\left(e^{\lambda_{2}}-1\right)}\int_{0}^{\infty }\frac{e^{-\alpha x+\lambda_{1}\Delta \left(x\right)}\left(e^{\lambda_{2}\Delta \left(x\right)}-1\right)}{{\left[1+e^{-\alpha x}\right]}^{2}}dx,\\ \; & =\frac{2\alpha \lambda_{1}}{\left(e^{\lambda_{1}}-1\right)\left(e^{\lambda_{2}}-1\right)}\int_{0}^{\infty }\frac{e^{-\alpha x+\left(\lambda_{1}+\lambda_{2}\right)\Delta \left(x\right)}}{{\left[1+e^{-\alpha x}\right]}^{2}}dx-\frac{1}{e^{\lambda_{2}}-1}. \end{array}$$

Let $u=1-e^{-\alpha x}$, then, $dx=du/(\alpha e^{-\alpha x})$, and $1+e^{-\alpha x}=1+\left(1-u\right)$, therefore,

$$\begin{array}{cc} R & =\frac{2\lambda_{1}}{\left(e^{\lambda_{1}}-1\right)\left(e^{\lambda_{2}}-1\right)}\int_{0}^{1}\frac{e^{\left(\lambda_{1}+\lambda_{2}\right)\left(\frac{u}{1+(1-u)}\right)}}{{\left[1+\left(1-u\right)\right]}^{2}}du-\frac{1}{e^{\lambda_{2}}-1}, \end{array}$$

(14) =2λ1(eλ1−1)(eλ2−1)∑i=0∞(λ1+λ2)ii!∫01ui[1+(1−u)]2+idu−1eλ2−1,=2λ1(eλ1−1)(eλ2−1)∑i,j=0∞i+j+1j(−1)j(λ1+λ2)ii!∫01ui(1−u)jdu−1eλ2−1,=2λ1(eλ1−1)(eλ2−1)∑i,j=0∞i+j+1j(−1)j(λ1+λ2)ii!B(i+1,j+1)−1eλ2−1,=∑i,j=0∞Ci,j*B(i+1,j+1)−1eλ2−1,

where Ci,j*=2λ1(λ1+λ2)i(−1)j(eλ1−1)(eλ2−1)i!i+j+1j. Notice that, in this case, the reliability *R* is independent of α.

### 3.1. Maximum Likelihood Estimation

Let $x_{1},x_{2},\ldots ,x_{n_{1}}$ be a random sample of size $n_{1}$ from PHLD1$\left(\alpha ,\lambda_{1}\right)$ and $y_{1},y_{2},\ldots ,y_{n_{2}}$ is an independent random sample of size $n_{2}$ from PHLD2$\left(\alpha ,\lambda_{2}\right)$. The log likelihood function is given by (15), here θ is given by $\theta ={\left(\alpha ,\lambda_{1},\lambda_{2}\right)}^{T}$.

(15) $$\begin{array}{cc} \log L & =\left(n_{1}+n_{2}\right)\log2+\left(n_{1}+n_{2}\right)\log\alpha +n_{1}\log\lambda_{1}+n_{2}\log\lambda_{2}-\alpha \sum_{i=1}^{n_{1}}x_{i}-\alpha \sum_{j=1}^{n_{2}}y_{j}\\ \; & -n_{1}\log\left(e^{\lambda_{1}}-1\right)-n_{2}\log\left(e^{\lambda_{2}}-1\right)-2\sum_{i=1}^{n_{1}}\log\left(1+e^{-\alpha x_{i}}\right)-2\sum_{j=1}^{n_{2}}\log\left(1+e^{-\alpha y_{j}}\right)\\ \; & +\lambda_{1}\sum_{i=1}^{n_{1}}\Delta \left(x_{i}\right)+\lambda_{2}\sum_{j=1}^{n_{2}}\Delta \left(y_{i}\right). \end{array}$$

The estimators of θ the $\hat{\theta }={\left(\hat{\alpha },{\hat{\lambda }}_{1},{\hat{\lambda }}_{2}\right)}^{T}$ can be obtained in similar way by solve the nonlinear Equations given (16)–(18) below,

(16) $$\begin{array}{cc} \frac{\partial L}{\partial \lambda_{1}} & =\frac{n_{1}}{\lambda_{1}}-\frac{n_{1}\;e^{\lambda_{1}}}{e^{\lambda_{1}}-1}+\sum_{i=1}^{n_{1}}\frac{1-e^{-\alpha x_{i}}}{1+e^{-\alpha x_{i}}}, \end{array}$$

(17) $$\begin{array}{cc} \frac{\partial L}{\partial \lambda_{2}} & =\frac{n_{2}}{\lambda_{2}}-\frac{n_{2}\;e^{\lambda_{2}}}{e^{\lambda_{2}}-1}+\sum_{j=1}^{n_{2}}\frac{1-e^{-\alpha y_{j}}}{1+e^{-\alpha y_{j}}}, \end{array}$$

(18) $$\begin{array}{cc} \frac{\partial L}{\partial \alpha } & =\frac{n_{1}+n_{2}}{\alpha }-\sum_{i=1}^{n_{1}}x_{i}-\sum_{j=1}^{n_{2}}y_{j}+2\sum_{i=1}^{n_{1}}\frac{x_{i}\;e^{-\alpha x_{i}}}{1+e^{-\alpha x_{i}}}+2\sum_{j=1}^{n_{2}}\frac{y_{j}\;e^{-\alpha y_{j}}}{1+e^{-\alpha y_{j}}},\\ \; & +2\lambda_{1}\sum_{i=1}^{n_{1}}\frac{x_{i}\;e^{-\alpha x_{i}}}{{\left(1+e^{-\alpha x_{i}}\right)}^{2}}+2\lambda_{2}\sum_{j=1}^{n_{2}}\frac{y_{j}\;e^{-\alpha y_{j}}}{{\left(1+e^{-\alpha y_{j}}\right)}^{2}}. \end{array}$$

Hence, the maximum likelihood estimator of R(θ) in (14) can be computed as $\hat{R}\left(\hat{\theta }\right)$.

### 3.2. Asymptotic Distribution and Confidence Intervals

In this subsection, we derived the asymptotic distribution of $\hat{\theta }={\left(\hat{\alpha },{\hat{\lambda }}_{1},{\hat{\lambda }}_{2}\right)}^{T}$, the asymptotic distribution of $\hat{R}$, then the asymptotic confidence intervals of *R*. The Fisher information matrix is I(θ)=−E[J(θ)], where $J\left(\theta \right)=\frac{\partial^{2}L}{\partial \theta \partial \theta^{T}}$, therefore,

$$I\left(\theta \right)=\left(\begin{array}{ccc} I_{\alpha \alpha } & I_{\alpha \lambda_{1}} & I_{\alpha \lambda_{2}}\\ I_{\lambda_{1}\alpha } & I_{\lambda_{1}\lambda_{1}} & I_{\lambda_{1}\lambda_{2}}\\ I_{\lambda_{2}\alpha } & I_{\lambda_{2}\lambda_{1}} & I_{\lambda_{2}\lambda_{2}} \end{array}\right)$$

The elements of J(θ) are given by

$$\begin{array}{cc} \frac{\partial^{2}L}{\partial \lambda_{1}^{2}} & =-\frac{n_{1}}{\lambda_{1}^{2}}-\frac{e^{\lambda }n_{1}}{e^{\lambda_{1}}-1}+\frac{e^{2\lambda_{1}}n_{1}}{{\left(e^{\lambda_{1}}-1\right)}^{2}},\\ \frac{\partial^{2}L}{\partial \lambda_{2}^{2}} & =-\frac{n_{2}}{\lambda_{2}^{2}}-\frac{e^{\lambda_{2}}n_{2}}{e^{\lambda_{2}}-1}+\frac{e^{2\lambda_{2}}n_{2}}{{\left(e^{\lambda_{2}}-1\right)}^{2}},\\ \frac{\partial^{2}L}{\partial \lambda_{1}\alpha } & =\sum_{i=1}^{n_{1}}\frac{2x_{i}\;e^{-\alpha x_{i}}}{{\left(1+e^{-\alpha x_{i}}\right)}^{2}},\\ \frac{\partial^{2}L}{\partial \lambda_{2}\alpha } & =\sum_{j=1}^{n_{2}}\frac{2y_{j}\;e^{-\alpha y_{j}}}{{\left(1+e^{-\alpha y_{j}}\right)}^{2}},\\ \frac{\partial^{2}L}{\partial \alpha^{2}} & =-\frac{n_{1}+n_{2}}{\alpha^{2}}-2\sum_{i=1}^{n_{1}}\frac{x_{i}^{2}\;e^{-\alpha x_{i}}}{\left(1+e^{-\alpha x_{i}}\right)}-2\sum_{j=1}^{n_{2}}\frac{y_{j}^{2}\;e^{-\alpha y_{j}}}{\left(1+e^{-\alpha y_{j}}\right)}+2\sum_{i=1}^{n_{1}}\frac{x_{i}^{2}\;e^{-2\alpha x_{i}}}{{\left(1+e^{-\alpha x_{i}}\right)}^{2}}+2\sum_{j=1}^{n_{2}}\frac{y_{j}^{2}\;e^{-2\alpha y_{j}}}{{\left(1+e^{-\alpha y_{j}}\right)}^{2}}\\ \; & -2\lambda_{1}\sum_{i=1}^{n_{1}}\frac{x_{i}^{2}\;e^{-\alpha x_{i}}}{{\left(1+e^{-\alpha x_{i}}\right)}^{2}}+4\lambda_{1}\sum_{i=1}^{n_{1}}\frac{x_{i}^{2}\;e^{-2\alpha x_{i}}}{{\left(1+e^{-\alpha x_{i}}\right)}^{3}}-2\lambda_{2}\sum_{j=1}^{n_{2}}\frac{y_{j}^{2}\;e^{-\alpha y_{j}}}{{\left(1+e^{-\alpha y_{j}}\right)}^{2}}+4\lambda_{2}\sum_{j=1}^{n_{2}}\frac{y_{j}^{2}\;e^{-2\alpha y_{j}}}{{\left(1+e^{-\alpha y_{j}}\right)}^{3}}, \end{array}$$

thus, the elements of *I* are given below by applying the Lemma 1.

$$\begin{array}{cc} I_{\lambda_{1}\lambda_{1}} & =-\frac{n_{1}}{\lambda_{1}^{2}}-\frac{e^{\lambda }n_{1}}{e^{\lambda_{1}}-1}+\frac{e^{2\lambda_{1}}n_{1}}{{\left(e^{\lambda_{1}}-1\right)}^{2}},\\ I_{\lambda_{2}\lambda_{2}} & =-\frac{n_{2}}{\lambda_{2}^{2}}-\frac{e^{\lambda_{2}}n_{2}}{e^{\lambda_{2}}-1}+\frac{e^{2\lambda_{2}}n_{2}}{{\left(e^{\lambda_{2}}-1\right)}^{2}},\\ I_{\alpha \lambda_{1}} & =-\frac{4n_{1}\alpha \lambda_{1}}{e^{\lambda_{1}}-1}\nabla \left(\alpha ,1,2,4,\lambda_{1}\right),\\ I_{\alpha \lambda_{2}} & =-\frac{4n_{2}\alpha \lambda_{2}}{e^{\lambda_{2}}-1}\nabla \left(\alpha ,1,2,4,\lambda_{2}\right),\\ I_{\alpha \alpha } & =\frac{\left(n_{1}+n_{2}\right)}{\alpha^{2}}+\frac{4n_{1}\alpha \lambda_{1}}{e^{\lambda_{1}}-1}\nabla \left(\alpha ,2,2,4,\lambda_{1}\right)+\frac{4n_{2}\alpha \lambda_{2}}{e^{\lambda_{2}}-1}\nabla \left(\alpha ,2,2,4,\lambda_{2}\right)+\frac{4n_{1}\alpha \lambda_{1}^{2}}{e^{\lambda_{1}}-1}\nabla \left(\alpha ,2,2,4,\lambda_{1}\right)\\ \; & -\frac{4n_{1}\alpha \lambda_{1}^{2}}{e^{\lambda_{1}}-1}\nabla \left(\alpha ,2,3,5,\lambda_{1}\right)+\frac{4n_{2}\alpha \lambda_{2}^{2}}{e^{\lambda_{2}}-1}\nabla \left(\alpha ,2,2,4,\lambda_{2}\right)-\frac{4n_{2}\alpha \lambda_{2}^{2}}{e^{\lambda_{2}}-1}\nabla \left(\alpha ,2,3,5,\lambda_{2}\right). \end{array}$$

**Lemma** **4.** *As $n_{1}\to \infty$ and $n_{2}\to \infty$ then, $\sqrt{n_{1}+n_{2}}\left(\hat{\theta }-\theta \right)\to N_{3}\left(0,I^{-1}\left(\theta \right)\right)$, where*

$$\lim_{n_{1},n_{2}\to \infty }\frac{1}{n_{1}+n_{2}}I^{-1}\left(\theta \right)=\left(\begin{array}{ccc} Var(\hat{\alpha }) & Cov(\hat{\alpha }\hat{\lambda_{1}}) & Cov(\hat{\alpha }\hat{\lambda_{2}})\\ Cov(\hat{\lambda_{1}}\hat{\alpha }) & Var(\hat{\lambda_{1}}) & Cov(\hat{\lambda_{1}}\hat{\lambda_{2}})\\ Cov(\hat{\lambda_{2}}\hat{\alpha }) & Cov(\hat{\lambda_{2}}\hat{\lambda_{1}}) & Var(\hat{\lambda_{2}}) \end{array}\right)$$

**Proof.**  follows from the asymptotic normality of MLE. □

To derive the asymptotic distribution of *R* as in similar way to the general case we compute the partial derivative of R(θ) in (14), remember that in this case *R* is independent of α, thus, $B\left(\theta \right)={\left(\frac{\partial R}{\partial \lambda_{1}},\frac{\partial R}{\partial \lambda_{2}}\right)}^{T}$ by considering the following Lemma 5.

**Lemma** **5.** Let x>0, $\Theta^{*}={\left(\alpha ,\lambda_{1},\lambda_{2},\delta_{1},\delta_{2},\delta_{3},\delta_{4}\right)}^{T}>0$, let,

$$\zeta^{*}\left(\Theta^{*}\right)=\int_{0}^{\infty }\frac{x^{\delta_{1}}\;e^{-\delta_{2}\alpha x+\left(\lambda_{1}+\lambda_{2}\right)\Delta \left(x\right)}{\left(1-e^{-\alpha x}\right)}^{\delta_{3}}}{{\left[1+e^{-\alpha x}\right]}^{\delta_{4}}}dx,$$

*then,*

$$\zeta^{*}\left(\Theta^{*}\right)=\sum_{i=1}^{\infty }\sum_{j=1}^{\infty }\phi_{i,j}^{*}\;B_{0,\delta_{1}}\left(\delta_{3}+i+1,\delta_{2}+j-1\right),$$

where ϕi,j*=(−1)δ1+j(λ1+λ2)iαδ1+1i!δ4+i+j−1j, in particular, for $\delta_{1}=1$ we have

$$\zeta^{*}\left(\Theta^{*}\right)=\sum_{i=1}^{\infty }\sum_{j=1}^{\infty }\phi_{i,j}^{*}\;B\left(3,j\right)\left(\psi_{0}\left(j\right)-\psi_{0}\left(j+3\right)\right),$$

with ϕi,j*=(−1)j+1(λ1+λ2)iα2i!δ4+i+j−1j.

**Proof.**  Let

$$\zeta^{*}\left(\Theta^{*}\right)=\int_{0}^{\infty }\frac{x^{\delta_{1}}\;e^{-\delta_{2}\alpha x+\left(\lambda_{1}+\lambda_{2}\right)\Delta \left(x\right)}{\left(1-e^{-\alpha x}\right)}^{\delta_{3}}}{{\left[1+e^{-\alpha x}\right]}^{\delta_{4}}}dx,$$

by the expansion of $e^{\left(\lambda_{1}+\lambda_{2}\right)\Delta \left(x\right)}$ and some algebraic simplification we have

$$\zeta^{*}\left(\Theta^{*}\right)=\sum_{i=0}^{\infty }\frac{{\left(\lambda_{1}+\lambda_{2}\right)}^{i}}{i!}\int_{0}^{\infty }\frac{x^{\delta_{1}}\;e^{-\delta_{2}\alpha x}\;{\left(1-e^{-\alpha x}\right)}^{\delta_{3}+i}}{{\left[1+e^{-\alpha x}\right]}^{\delta_{4}+i}}dx,$$

letting $u=1-e^{-\alpha x}$ and then expansion of the denominator, we get

ζ*(Θ*)=∑i=0∞(λ1+λ2)ii!(−1)δ1αδ1+1∫01logδ1(1−u)uδ3+i(1−u)δ2−1(1+(1−u))δ4+idu,=∑i=0∞∑j=0∞(λ1+λ2)ii!(−1)δ1+1αδ1+1δ4+i+j−1j∫01logδ1(1−u)uδ3+i(1−u)δ2+j−1du.

Thus,

$$\zeta^{*}\left(\Theta^{*}\right)=\sum_{i=1}^{\infty }\sum_{j=1}^{\infty }\phi_{i,j}^{*}\;B_{0,\delta_{1}}\left(\delta_{3}+i+1,\delta_{2}+j-1\right),$$

where ϕi,j*=(−1)δ1+j(λ1+λ2)iαδ1+1i!δ4+i+j−1j. □

From the above lemma we derive $\frac{\partial R}{\partial \lambda_{1}}$ and $\frac{\partial R}{\partial \lambda_{2}}$ as follows.

$$\begin{array}{cc} \frac{\partial R}{\partial \lambda_{2}} & =-\frac{2\alpha \lambda_{1}e^{\lambda_{2}}}{\left(e^{\lambda_{1}}-1\right){\left(e^{\lambda_{2}}-1\right)}^{2}}\int_{0}^{\infty }\frac{e^{-\alpha x+\left(\lambda_{1}+\lambda_{2}\right)\Delta \left(x\right)}}{{\left(1+e^{-\alpha x}\right)}^{2}}dx\\ \; & +\frac{2\alpha \lambda_{1}}{\left(e^{\lambda_{1}}-1\right)\left(e^{\lambda_{2}}\right)-1}\int_{0}^{\infty }\frac{\Delta \left(x\right)\;e^{-\alpha x+\left(\lambda_{1}+\lambda_{2}\right)\Delta \left(x\right)}}{{\left(1+e^{-\alpha x}-1\right)}^{2}}dx+\frac{e^{\lambda_{2}}}{{\left(e^{\lambda_{2}}-1\right)}^{2}}\\ \; & =-\frac{2\alpha \lambda_{1}e^{\lambda_{2}}}{\left(e^{\lambda_{1}}-1\right){\left(e^{\lambda_{2}}-1\right)}^{2}}\zeta^{*}\left(\alpha ,\lambda_{1},\lambda_{2},0,1,0,2\right)\\ \; & +\frac{2\alpha \lambda_{1}}{\left(e^{\lambda_{1}}-1\right)\left(e^{\lambda_{2}}-1\right)}\zeta^{*}\left(\alpha ,\lambda_{1},\lambda_{2},0,1,1,3\right)+\frac{e^{\lambda_{2}}}{{\left(e^{\lambda_{2}}-1\right)}^{2}}, \end{array}$$

$$\begin{array}{cc} \frac{\partial R}{\partial \lambda_{1}} & =\frac{2\alpha }{\left(e^{\lambda_{1}}-1\right)\left(e^{\lambda_{2}}-1\right)}\int_{0}^{\infty }\frac{e^{-\alpha x+\left(\lambda_{1}+\lambda_{2}\right)\Delta \left(x\right)}}{{\left(1+e^{-\alpha x}\right)}^{2}}dx\\ \; & -\frac{2\alpha \lambda_{1}e^{\lambda_{1}}}{\left(e^{\lambda_{1}}-1\right)\left(e^{\lambda_{2}}-1\right)}\int_{0}^{\infty }\frac{e^{-\alpha x+\left(\lambda_{1}+\lambda_{2}\right)\Delta \left(x\right)}}{{\left(1+e^{-\alpha x}\right)}^{2}}dx\\ \; & +\frac{2\alpha \lambda_{1}}{\left(e^{\lambda_{1}}-1\right)\left(e^{\lambda_{2}}-1\right)}\int_{0}^{\infty }\frac{\Delta \left(x\right)\;e^{-\alpha x+\left(\lambda_{1}+\lambda_{2}\right)\Delta \left(x\right)}}{{\left(1+e^{-\alpha x}\right)}^{2}}dx\\ \; & =\frac{2\alpha }{\left(e^{\lambda_{1}}-1\right)\left(e^{\lambda_{2}}-1\right)}\zeta^{*}\left(\alpha ,\lambda_{1},\lambda_{2},0,1,0,2\right)-\frac{2\alpha \lambda_{1}e^{\lambda_{1}}}{\left(e^{\lambda_{1}}-1\right)\left(e^{\lambda_{2}}-1\right)}\zeta^{*}\left(\alpha ,\lambda_{1},\lambda_{2},0,1,0,2\right)\\ \; & +\frac{2\alpha \lambda_{1}}{\left(e^{\lambda_{1}}-1\right)\left(e^{\lambda_{2}}-1\right)}\zeta^{*}\left(\alpha ,\lambda_{1},\lambda_{2},0,1,1,3\right). \end{array}$$

In similar way, we can obtain the asymptotic distribution of $\hat{R}\left(\theta \right)$ as

$$\sqrt{n_{1}+n_{2}}\left(\hat{R}-R\right)\to N_{3}\left(0,B^{T}\left(\theta \right)I^{-1}\left(\theta \right)B\left(\theta \right)\right),$$

hence, the asymptotic variance of $\hat{R}$ is computed as

$$\begin{array}{cc} Var\left(\hat{R}\right)=\frac{1}{n_{1}+n_{2}}B^{T}\left(\theta \right)I^{-1}\left(\theta \right)B\left(\theta \right) & ={\left(\frac{\partial R}{\partial \lambda_{1}}\right)}^{2}Var\left(\hat{\lambda_{1}}\right)+{\left(\frac{\partial R}{\partial \lambda_{2}}\right)}^{2}Var\left(\hat{\lambda_{2}}\right)\\ \; & +2\frac{\partial R}{\partial \lambda_{1}}\frac{\partial R}{\partial \lambda_{2}}Cov\left(\hat{\lambda_{1}}\hat{\lambda_{2}}\right). \end{array}$$

The 100(1−ϵ) asymptotic confidence interval for *R* can be constructed as

$$\hat{R}\pm Z_{\frac{\epsilon }{2}}\sqrt{Var(\hat{R})},$$

where $Z_{\frac{\epsilon }{2}}$ is the upper $\frac{\epsilon }{2}$ quantile of the standard normal distribution. Moreover, we can use the bootstrap confidence interval preferably for moderate sample sizes, the computation of the bootstrap confidence interval follows similarly to the steps given in Section 2.3.

## 4. Bayes Estimation of *R*

In this section, we discuss the Bayes estimation of *R* in general case and the Bayes estimation of *R* with common scale parameter α. We employ the use of the Bayesian estimation to estimate *R* under various loss functions. The point estimators $\hat{\vartheta }$ are derived from the posterior distributions given the sample data. The estimator that minimizes the square error loss function (SEL) for the assumed prior distribution is ${\left(\hat{\vartheta }-\vartheta \right)}^{2}$ which is the posterior mean, here, we compute ${\hat{R}}_{SEL}=\frac{1}{N-M}\sum_{i=M+1}^{N}R^{\left(i\right)}$. The absolute error loss function (AEL), $|\hat{\vartheta }-\vartheta |$ for the assumed prior distribution is minimizes by the posterior median as ${\hat{R}}_{AEL}$. The maximum a posteriori (MAP) can be used to obtain the estimators when there is no loss function, it depends on the likelihood function and prior distribution, that makes it closely related to maximum likelihood, it is the value that maximizes the posterior distribution i.e., the mode. The linear exponential loss function (LINEX) with parameters *c* is defined by $\left(e^{c(\hat{\vartheta }-\vartheta )}-c\left(\hat{\vartheta }-\vartheta \right)-1\right)$ and we can minimized by the estimator ${\hat{R}}_{LIN}=\frac{-1}{c}\log\left[\frac{1}{N-M}\sum_{i=M+1}^{N}e^{cR^{\left(i\right)}}\right]$, the sign of the parameter c reflect the direction of asymmetry, while its magnitude reflect the degree of the asymmetry. The general entropy loss function (GEL) [42] is ${\left(\frac{\hat{\vartheta }}{\vartheta }\right)}^{q}-q\left(\frac{\hat{\vartheta }}{\vartheta }\right)-1$, and its minimized by ${\hat{R}}_{GEL}={\left[\frac{1}{N-M}\sum_{i=M+1}^{N}{\left(R^{\left(i\right)}\right)}^{-q}\right]}^{-\frac{1}{q}}$. Moreover, the highest posterior density (HPD) credible interval for *R* is constructed. N is the number of iterations and M is the burn in.

### 4.1. Bayes Estimation of *R* in General Case

Let $x_{1},x_{2},\ldots ,x_{n_{1}}$ is an independent random sample of size $n_{1}$ from PHLD1$\left(\alpha_{1},\lambda_{1}\right)$ and $y_{1},y_{2},\ldots ,y_{n_{2}}$ is an independent random sample of size $n_{2}$ from PHLD2$\left(\alpha_{2},\lambda_{2}\right)$. Let assumed that $\alpha_{1},\;\alpha_{2},\;\lambda_{1},\;\lambda_{2}$ are independent and follow gamma density function $Gamma(a_{1},b_{1}),$$Gamma(a_{2},b_{2}),$$Gamma(a_{3},b_{3})$, and $Gamma(a_{4},b_{4})$, respectively. Then the joint density of the data, $\alpha_{1},\;\alpha_{2},\;\lambda_{1},\;\lambda_{2}$ is given by

$$\ell \left(data;\alpha_{1},\alpha_{2},\lambda_{1},\lambda_{2}\right)=L\left(\alpha_{1},\alpha_{2},\lambda_{1},\lambda_{2}|data\right)\pi_{1}\left(\alpha_{1}\right)\pi_{2}\left(\alpha_{2}\right)\pi_{3}\left(\lambda_{1}\right)\pi_{4}\left(\lambda_{2}\right),$$

where $\pi_{i}\left(.\right),\;i=1,2,3,4,$ are the gamma prior density for $\alpha_{1},\;\alpha_{2},\;\lambda_{1}$ and $\lambda_{2}$ respectively. Thus, the joint posterior density of $\alpha_{1},\;\alpha_{2},\;\lambda_{1},\;\lambda_{2}$ given the data sets is given by

(19) $$P\left(\alpha_{1},\alpha_{2},\lambda_{1},\lambda_{2}|data\right)=\frac{\ell (data;\alpha_{1},\alpha_{2},\lambda_{1},\lambda_{2})}{\int \int \int \int \ell \left(data;\alpha_{1},\alpha_{2},\lambda_{1},\lambda_{2}\right)d\alpha_{1}d\alpha_{2}d\lambda_{1}d\lambda_{2}}.$$

The above Equation (19) cannot be expressed in a closed form, therefore, we employ the Gibbs sampling technique to compute the Bayes estimate of *R* under various measures and an approximate 100(1−ϵ)% credible interval of *R*. The marginal posterior densities of $\alpha_{1},\;\alpha_{2},\;\lambda_{1}$ and $\lambda_{2}$ are:

(20) $$\begin{array}{cc} P_{1}\left(\alpha_{1}|data\right) & \propto \alpha_{1}^{n_{1}+a_{1}-1}\;e^{-\alpha_{1}\left(b_{1}+\sum_{i=1}^{n_{1}}x_{i}\right)+\lambda_{1}\sum_{i=1}^{n_{1}}\Delta_{1}\left(x_{i}\right)}\;{\left(\prod_{i=1}^{n_{1}}{\left(1+e^{-\alpha_{1}x_{i}}\right)}^{2}\right)}^{-1}, \end{array}$$

(21) $$\begin{array}{cc} P_{2}\left(\alpha_{2}|data\right) & \propto \alpha_{2}^{n_{2}+a_{2}-1}\;e^{-\alpha_{2}\left(b_{2}+\sum_{j=1}^{n_{2}}y_{j}\right)+\lambda_{2}\sum_{j=1}^{n_{2}}\Delta_{2}\left(y_{j}\right)}\;{\left(\prod_{j=1}^{n_{2}}{\left(1+e^{-\alpha_{2}y_{j}}\right)}^{2}\right)}^{-1}, \end{array}$$

(22) $$\begin{array}{cc} P_{3}\left(\lambda_{1}|data\right) & \propto \lambda_{1}^{n_{1}+a_{3}-1}\;e^{-\lambda_{1}\left(b_{3}-\sum_{i=1}^{n_{1}}\Delta_{1}\left(x_{i}\right)\right)}\;{\left(e^{\lambda_{1}}-1\right)}^{-n_{1}}, \end{array}$$

(23) $$\begin{array}{cc} P_{4}\left(\lambda_{2}|data\right) & \propto \lambda_{2}^{n_{2}+a_{4}-1}\;e^{-\lambda_{2}\left(b_{4}-\sum_{j=1}^{n_{2}}\Delta_{2}\left(y_{j}\right)\right)}\;{\left(e^{\lambda_{2}}-1\right)}^{-n_{2}}. \end{array}$$

The marginal conditional distributions obtained from the posterior distribution $P_{i}$ in (20)–(23) are not straightforward, they are not from well-known distributions, so we are going to obtain samples by applying the Metropolis– Hastings algorithm, see [43,44,45], we take our proposal distribution to be a normal distribution. In general, we consider the Gibbs sampling technique to generate samples from the posterior distributions, then to compute the Bayes estimators of *R* with respect to some loss functions. We can obtain the highest posterior density (HPD) interval for *R*. The step-by-step Gibbs sampling algorithm is given below:

- Step 1: Start with initial guess $\left(\alpha_{1}^{\left(0\right)},\alpha_{2}^{\left(0\right)},\lambda_{1}^{\left(0\right)},\lambda_{2}^{\left(0\right)}\right)$
- Step 2: Set t=1
- Step 3: Use the Metropolis–Hastings algorithm to generate $\alpha_{1}^{\left(t\right)}$ from $P_{1}$ and $\lambda_{1}^{\left(t\right)}$ from $P_{3}$
- Step 4: Use the Metropolis–Hastings algorithm to generate $\alpha_{2}^{\left(t\right)}$ from $P_{2}$ and $\lambda_{2}^{\left(t\right)}$ from $P_{4}$
- Step 5: Compute $R^{\left(t\right)}$ from Equation (7)
- Step 6: Set t=t+1
- Step 7: Repeat step 3 to 6, *T* times.

For sufficiently large value of *T*, we can have an approximate of $R_{SEL},R_{AEL},R_{MAP},R_{LIN},$ and $R_{GEL}.$ An approximate 100(1−ϵ)% credible interval of *R* from SEL can computed by using the procedure provided by [46] as the shortest distance of the intervals of $\left({\hat{R}}^{\left(1\right)},{\hat{R}}^{(1-\epsilon )T}\right),\;\left({\hat{R}}^{2},{\hat{R}}^{\left(1-\epsilon )(T+1\right)}\right),\;\ldots ,\left({\hat{R}}^{\epsilon T},{\hat{R}}^{T}\right)$.

### 4.2. Bayes Estimation of *R* with Common Scale Parameter α

Let $x_{1},x_{2},\ldots ,x_{n_{1}}$ is an independent random sample of $n_{1}$ size from PHLD1$\left(\alpha ,\lambda_{1}\right)$ and $y_{1},y_{2},\ldots ,y_{n_{2}}$ is an independent random sample of size $n_{2}$ from PHLD2$\left(\alpha ,\lambda_{2}\right)$. Let assumed that $\alpha ,\lambda_{1},\lambda_{2}$ are independent with gamma density function $Gamma\left(a_{1},b_{1}\right),\;Gamma\left(a_{2},b_{2}\right),$ and $Gamma(a_{3},b_{3})$, respectively. Then the joint density of the data, $\alpha ,\;\lambda_{1},\;\lambda_{2}$ is given by

$$\ell \left(data;\alpha ,\lambda_{1},\lambda_{2}\right)=L\left(\alpha ,\lambda_{1},\lambda_{2}|data\right)\pi_{1}\left(\alpha \right)\pi_{2}\left(\lambda_{1}\right)\pi_{3}\left(\lambda_{2}\right),$$

where $\pi_{i}\left(.\right),\;i=1,2,3,$ are the gamma prior density for $\alpha ,\lambda_{1}$ and $\lambda_{2}$ respectively. Thus, the joint posterior density of $\alpha ,\lambda_{1},\lambda_{2}$ given the data sets is given by

(24) $$P\left(\alpha ,\lambda_{1},\lambda_{2}|data\right)=\frac{\ell (data;\alpha ,\lambda_{1},\lambda_{2})}{\int \int \int \ell \left(data;\alpha ,\lambda_{1},\lambda_{2}\right)d\alpha d\lambda_{1}d\lambda_{2}}.$$

The above Equation (24) is required to apply the Gibbs sampling technique to obtain the Bayes estimates of $\alpha ,\lambda_{1},\lambda_{2}$ to compute *R* and its credible interval. The posterior densities of $\alpha ,\lambda_{1}$ and $\lambda_{2}$ are:

(25) $$\begin{array}{cc} P_{1}\left(\alpha |data\right) & \propto \frac{\alpha^{n_{1}+n_{2}+a_{1}-1}\;e^{-\alpha \left(b_{1}+\sum_{i=1}^{n_{1}}x_{i}+\sum_{j=1}^{n_{2}}y_{j}\right)+\lambda_{1}\sum_{i=1}^{n_{1}}\left(x_{i}\right)+\lambda_{2}\sum_{j=1}^{n_{2}}\left(y_{j}\right)}}{\prod_{i=1}^{n_{1}}{\left(1+e^{-\alpha x_{i}}\right)}^{2}\prod_{j=1}^{n_{2}}{\left(1+e^{-\alpha y_{j}}\right)}^{2}}, \end{array}$$

(26) $$\begin{array}{cc} P_{2}\left(\lambda_{1}|data\right) & \propto \lambda_{1}^{n_{1}+a_{2}-1}\;e^{-\lambda_{1}\left(b_{2}-\sum_{i=1}^{n_{1}}\Delta \left(x_{i}\right)\right)}\;{\left(e^{\lambda_{1}}-1\right)}^{-n_{1}}, \end{array}$$

(27) $$\begin{array}{cc} P_{3}\left(\lambda_{2}|data\right) & \propto \lambda_{2}^{n_{2}+a_{3}-1}\;e^{-\lambda_{2}\left(b_{3}-\sum_{j=1}^{n_{2}}\Delta \left(y_{j}\right)\right)}\;{\left(e^{\lambda_{2}}-1\right)}^{-n_{2}}. \end{array}$$

Here, the posterior distributions $P_{i}$ in (25)–(27) are not from well-known distributions, therefore we apply the Gibbs sampling technique to generate samples from the posterior distributions as in the Section 4.1 above, then to compute the Bayes estimators of *R* with respect to some loss function, and the HPD credible interval for *R*. The steps are given below:

1. Step 1: Start with initial guess $\left(\alpha^{\left(0\right)},\lambda_{1}^{\left(0\right)},\lambda_{2}^{\left(0\right)}\right)$
2. Step 2: Set t=1
3. Step 3: Use the Metropolis–Hastings algorithm to generate $\lambda_{1}^{\left(t\right)}$ from $P_{2}$ and $\lambda_{2}^{\left(t\right)}$ from $P_{3}$
4. Step 4: Use the Metropolis–Hastings algorithm to generate $\alpha^{\left(t\right)}$ from $P_{1}$
5. Step 5: Compute $R^{\left(t\right)}$ from Equation (14)
6. Step 6: Set t=t+1
7. Step 7: Repeat step 3 to 6, *T* times.

For sufficiently large value of *T* the approximate $R_{SEL},\;R_{AEL},\;R_{MAP},\;R_{LIN},\;R_{GEL},$ and the HPD credible interval of *R* can be computed from the resulting sampling as described in Section 4.1.

## 5. Simulation

In this section, Monte Carlo simulation was used to examine the performance of the different estimators discussed. Simulated samples were generated using different values of parameters from independent PHLD$1(\alpha_{1},\lambda_{1})$ and PHLD$2(\alpha_{2},\lambda_{2})$ of sizes say $n_{1}$ and $n_{2}$ respectively, using Equation (2). We consider the cases when $n_{1}=n_{2}$, $n_{1}>n_{2}$ and $n_{1}<n_{2}$ as (20,20),(40,30),(40,50) and (60,60). The simulation studies were conducted using 1000 samples from PHLD1 and PHLD2. The MLE and the 95% asymptotic confidence interval (AS${}_{CI}$) from the expected information matrix were computed, also the percentile bootstrap (B${}_{p}$) and student’s bootstrap B${}_{t}$ confidence interval was computed based on B=1000 replications. The Bayes estimator of *R* was computed under various loss functions with M=1000 iterations by considering the first 10% as a burn-in, also the 95% HPD credible interval $HPD_{CI}$ is observed based on SEL by the package HDInterval[47] in R-software. For the estimators based on LINEX and GEL, we take c=2 and q=3 respectively. We discussed the Bias, mean square error (MSE), and confidence intervals with their coverage probability CP of *R* based on the various estimation techniques. All the computations were performed using R-software[48]. The resulting simulation obtained were presented in Table 1 and Table 2. Observe from these tables that: (i) the MSE decreases as the sample sizes increases in both the estimators; (ii) the MSE of *R* based on $R_{SEL},\;R_{AEL}$ and $R_{LIN}$ are more closer in most cases; (iii) based on our choice of q=3 the bias of the $R_{GEL}$ is negative in the majority the of cases; (iv) the average length of confidence interval (ALCI) decreases as the sample size increases in all the techniques; (v) the MLE has larger ALCI for smaller sample size and smaller ALCI for the largest size in most cases; (vi) the Bayes estimators has averagely smaller size of confidence interval as compared in all the cases; (vii) the $B_{P}$ and $B_{t}$ performance is quite good, their CI appear almost the same for all the cases, and their coverage probability covers the nominal sizes in most cases both for small and large sample size; (viii) in general, both the estimation technique and confidence interval were quite sufficient and can be used to analyzed stress strength data from PHLD, but for smaller sample size we recommend the used of bootstrap $B_{p}$ and $B_{t}$ for estimation of confidence interval.

## 6. Real Data Study

In this section, we provide two real data applications to demonstrate how the proposed estimation techniques can be applied in practice. We computed *R* by maximum likelihood, and Bayes estimation under the various loss functions discussed, also the four confidence intervals studied are obtained. The goodness of fit statistic, called Kolmogorov–Smirnov (KS), was used to demonstrate how good the models fit the data sets by the proposed techniques. We consider the B=1000 replication for the bootstrap confidence interval and M=10,000 for the Bayes estimation and the first 10% burn-in. For the $R_{LIN}$ we consider c=2 and q=3 for the $R_{GEL}$.

### 6.1. Real Data Study 1

This is the strength data measured in GPA, for single carbon fibers and impregnated 1000-carbon fiber tows. Single fibers were tested under tension at gauge lengths of 1, 10, 20, and 50 mm, also impregnated tows of 1000 fibers were tested at gauge lengths of 20, 50, 150, and 300 mm, some of these data set were studied in the stress strength analysis by [27,49]. Here are the single fibers of 20 mm (data1) and 10 mm (data2) in gauge lengths. The data was provided by [50] also analyzed by [51,52,53].

Data1: ($n_{1}=69$) 0.312, 0.314, 0.479, 0.552, 0.700, 0.803, 0.861, 0.865, 0.944, 0.958, 0.966, 0.997, 1.006, 1.021, 1.027, 1.055, 1.063, 1.098, 1.140, 1.179, 1.224, 1.240, 1.253, 1.270, 1.272, 1.274, 1.301, 1.301, 1.359, 1.382, 1.382, 1.426, 1.434, 1.435, 1.478, 1.490, 1.511, 1.514, 1.535, 1.554, 1.566, 1.570, 1.586, 1.629, 1.633, 1.642, 1.648, 1.684, 1.697, 1.726, 1.770, 1.773, 1.800, 1.809, 1.818, 1.821, 1.848, 1.880, 1.954, 2.012, 2.067, 2.084, 2.090, 2.096, 2.128, 2.233, 2.433, 2.585, 2.585, and

Data2: ($n_{2}=63$) 0.101, 0.332, 0.403, 0.428, 0.457, 0.550, 0.561, 0.596, 0.597, 0.645, 0.654, 0.674, 0.718, 0.722, 0.725, 0.732, 0.775, 0.814, 0.816, 0.818, 0.824, 0.859, 0.875, 0.938, 0.940, 1.056, 1.117, 1.128, 1.137, 1.137, 1.177, 1.196, 1.230, 1.325, 1.339, 1.345, 1.420, 1.423, 1.435, 1.443, 1.464, 1.472, 1.494, 1.532, 1.546, 1.577, 1.608, 1.635, 1.693, 1.701, 1.737, 1.754, 1.762, 1.828, 2.052, 2.071, 2.086, 2.171, 2.224, 2.227, 2.425, 2.595, 3.220.

**Figure 1:**
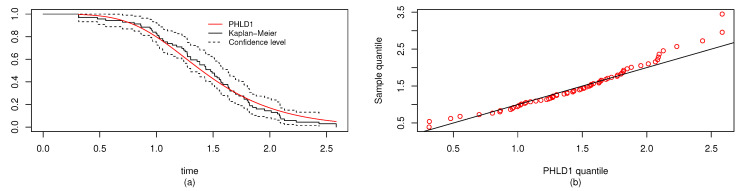
(**a**) fitted Poisson half logistic random variables (PHLD)1 survival function, and (**b**) quantile–quantile plots of the PHLD1 for data1 of the real data study 1.

**Figure 2:**
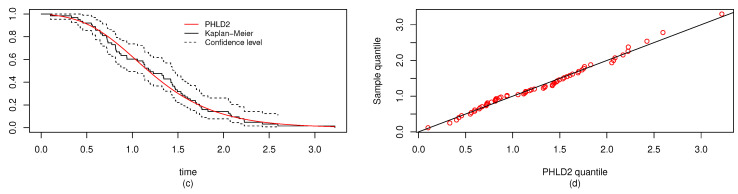
(**c**) Fitted PHLD2 survival function, and (**d**) quantile–quantile plots of the PHLD2 for data2 of the real data study 1.

**Figure 3:**
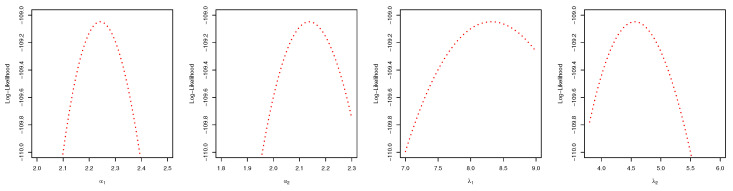
Plots of the profile log-likelihood for each parameter $\alpha_{1}$ (**left**), $\alpha_{2}$ (**middle-left**), $\lambda_{1}$ (**middle-right**), $\lambda_{2}$ (**right**) for the real data study 1.

**Figure 4:**
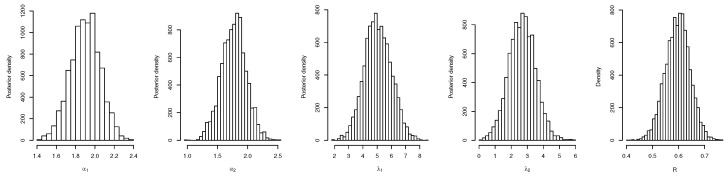
Plots of the posterior densities of each parameter and the density of *R* for the real data study 1; $\alpha_{1}$ (**left**), $\alpha_{2}$ (**middle-left**), $\lambda_{1}$ (**middle**), $\lambda_{2}$ (**middle-right**) and *R* (**right**).

**Figure 5:**
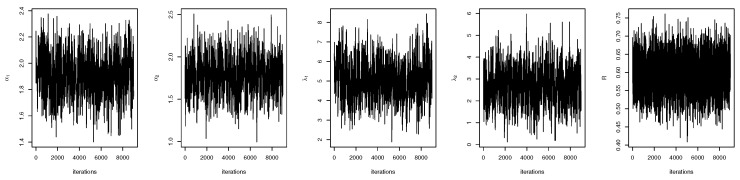
Iterations obtained from the Gibbs and Metropolis–Hastings algorithms for each parameter and *R* for the real data study 1; $\alpha_{1}$ (**left**), $\alpha_{2}$ (middle-**left**), $\lambda_{1}$ (**middle**), $\lambda_{2}$ (**middle-right**) and *R* (**right**).

For both the MLE and Bayes techniques the estimated parameters, KS with *p*-values are presented, the log-likelihood (L) for the MLEs are given in Table 3. Table 4 provides the estimated values of *R*, the confidence intervals, and their length. The confidence intervals obtained include the asymptotic confidence interval, $B_{p}$, $B_{t}$, and the HPD credible interval. From the table, all the *R* computed were almost similar except that the MAP and MLE are very closed. The confidence intervals are quite good and almost the same length in all the techniques. Table 3 shown that PHLD gives a better fit for both data sets by considering the KS values. Based on the MLE, Figure 1a and Figure 2c show the empirical and the fitted PHLD survival functions for the data1 and data2, it can be seen graphically how the PHLD survival curve fitted the empirical curve, indicating how good PHLD represented the two data sets. Figure 1b and Figure 2d show the quantile–quantile plots for data1 and data2, where almost all of the quantile points lie on the straight line this also shows how good PHLD represent the data sets. Figure 3 is the profile log-likelihood for each parameter, it is clear from the curves that the maximized log-likelihood function has a unique value. Figure 4 is the posterior densities of each parameter and the density of *R* base on the iterations obtained from the Bayes estimation, also showing how good the Bayes estimation performed and both the densities go to the true posterior densities of their parameters. Figure 5 is the iterations obtained from the Gibbs and Metropolis–Hastings algorithms of each parameter and computed *R* from the Bayes technique, notice that for each parameter the iterated sample values were centered to their mean and the iterations converge to their true population density in each of the parameters within the first few iterations. This demonstrated the performance of the Bayes estimators of the parameters of PHLD. From this application, we can see that PHLD is a good model for reliability studies.

### 6.2. Real Data Study 2

In other fields of studies, *R* is considered an important measure to study the difference between the two populations. Here, we analyzed the data sets studied by [54,55,56,57] in the estimation of the stress strength parameter. The data set consists of the waiting times before the service of the customers of two banks A (data1) and B (data2), the data1 were also discussed by [58].

Data1: ($n_{1}=100$) 0.8, 0.8, 1.3, 1.5, 1.8, 1.9, 1.9, 2.1, 2.6, 2.7, 2.9, 3.1, 3.2,3.3, 3.5, 3.6,4.0, 4.1, 4.2, 4.2, 4.3, 4.3, 4.4, 4.4, 4.6, 4.7, 4.7, 4.8, 4.9, 4.9, 5.0, 5.3, 5.5, 5.7, 5.7, 6.1, 6.2, 6.2, 6.2, 6.3, 6.7, 6.9, 7.1, 7.1, 7.1, 7.1, 7.4, 7.6, 7.7, 8.0, 8.2, 8.6, 8.6, 8.6, 8.8, 8.8, 8.9, 8.9, 9.5, 9.6, 9.7, 9.8, 10.7, 10.9, 11.0, 11.0, 11.1, 11.2, 11.2, 11.5, 11.9, 12.4, 12.5, 12.9, 13.0, 13.1, 13.3, 13.6, 13.7, 13.9, 14.1, 15.4, 15.4, 17.3, 17.3, 18.1, 18.2, 18.4, 18.9, 19.0, 19.9, 20.6, 21.3, 21.4, 21.9, 23.0, 27.0, 31.6, 33.1, 38.5, and

Data2: ($n_{2}=60$) 0.1, 0.2, 0.3, 0.7, 0.9, 1.1, 1.2, 1.8, 1.9, 2.0, 2.2, 2.3, 2.3, 2.3, 2.5, 2.6, 2.7, 2.7, 2.9, 3.1, 3.1, 3.2, 3.4, 3.4, 3.5, 3.9, 4.0, 4.2, 4.5, 4.7, 5.3, 5.6, 5.6, 6.2, 6.3, 6.6, 6.8, 7.3, 7.5, 7.7, 7.7, 8.0, 8.0, 8.5, 8.5, 8.7, 9.5, 10.7, 10.9, 11.0, 12.1, 12.3, 12.8, 12.9, 13.2, 13.7, 14.5, 16.0, 16.5, 28.0.

**Figure 6:**
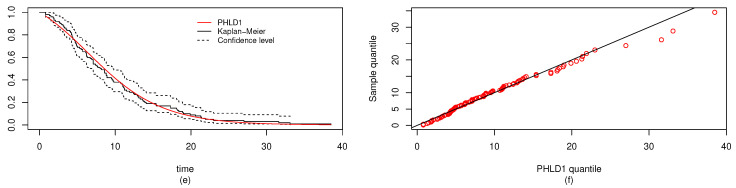
(**e**) Fitted PHLD1 survival function, and (**f**) quantile-quantile plots of the PHLD1 for data1 of the real data study 2.

**Figure 7:**
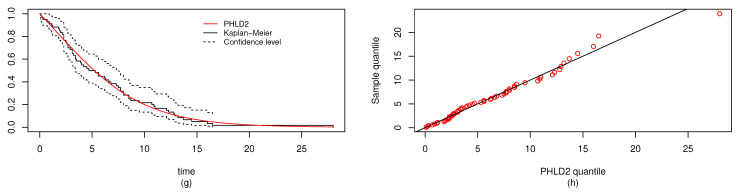
(**g**) Fitted PHLD2 survival function, and (**h**) quantile-quantile plots of the PHLD2 for data2 of the real data study 2.

**Figure 8:**
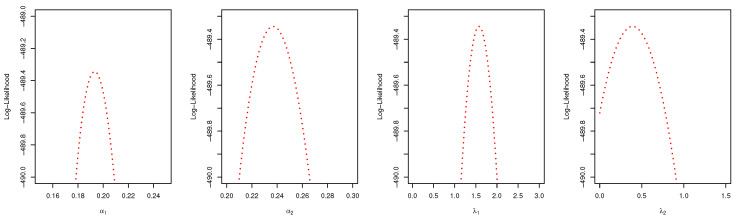
Plots of the profile log-likelihood for each parameter $\alpha_{1}$ (**left**), $\alpha_{2}$ (**middle-left**), $\lambda_{1}$ (**middle-right**), $\lambda_{2}$ (**right**) for the real data study 2.

**Figure 9:**
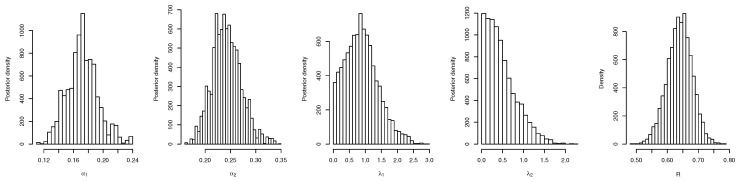
Plots of the posterior densities of each parameter and the density of *R* for the real data study 2; $\alpha_{1}$ (**left**), $\alpha_{2}$ (**middle-left**), $\lambda_{1}$ (**middle**), $\lambda_{2}$ (**middle-right**) and *R* (**right**).

**Figure 10:**
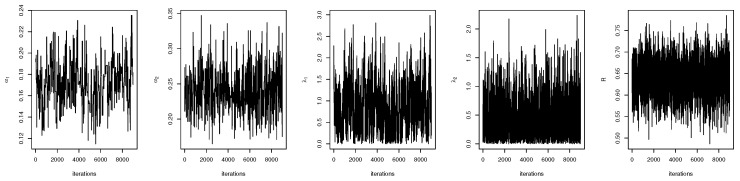
Iterations obtained from the Gibbs and Metropolis–Hastings algorithms for each parameter and R for the real data study 2; $\alpha_{1}$ (**left**), $\alpha_{2}$ (**middle-left**), $\lambda_{1}$ (**middle**), $\lambda_{2}$ (**middle-right**) and *R* (**right**).

In a similar way, the MLEs and the Bayes estimators of the parameters, KS with *p*-values and log-likelihood (L) of the MLE are computed and given in Table 5. The estimated values of *R*, the confidence intervals, and the length of the confidence interval computed from the various techniques are provided in Table 6. Observed that all the *R* computed were closer and the confidence intervals are quite good. It is clear from Table 5 that PHLD provides a good fit for both data sets by considering the KS values. Based on the MLE, Figure 6 and Figure 7 show the fitted PHLD survival function (e), (g) and quantile-quantile plots (f), (h) for the data1 and data2 to illustrate how PHLD represents the two data set. Figure 8 is the profile log-likelihood for each parameter showing that the Log-likelihood is unique. We also provided in Figure 9 the posterior densities of each parameter and the density of *R* base on the iterations from the Bayes estimation, whereas Figure 10 shows the Gibbs and Metropolis–Hastings algorithms iterations of each parameter and the computed *R*; this is indicating the good performance of the Bayes estimators. From this illustration, it’s quite clear that PHLD can be a good candidate in stress strength reliability analysis.

## 7. Conclusions

The estimation of the stress–strength parameter *R* when the random variables *X* and *Y* are independent Poisson half logistic distributions are provided. We have addressed the case in general and when the scale parameter is common. The point and interval estimation of *R* was discussed; these include the maximum likelihood estimation of *R* and its asymptotic confidence interval, percentile bootstrap and student’s bootstrap confidence interval; Bayes estimation of *R* is computed under the square error loss function, absolute error loss function, linear exponential error loss function, generalized entropy error loss function, and maximum a posteriori, also the credible interval based on the square error loss function is obtained. We examine by simulation studies the proposed point and interval estimates, and they work very well for various samples sizes as discussed by their MSE and the confidence intervals; the MSE decreases as the sample increases in both techniques, and based on the simulation result we recommend the use of the bootstrap for estimating the confidence interval of very small size. We used two real data studies to demonstrate the performance of the two estimators of *R* in practical applications, the MLE and the BE estimators goodness of fit for each real data were examined by KS statistic and the result was sufficient and satisfactory, the Gibbs and Metropolis–Hastings iterations in the Bayes estimators converge to their true parameter within the first few iterations in both data sets. In each of the two real data studies the estimates of *R* obtained from the MLE and BE techniques were closely identical. We hoped that the PHLD will be a very useful tool in stress–strength reliability studies. Based on our results, we suggested that the Bayesian estimation of the model can be further discussed under different priors, also the analysis of the MLE and the Bayes estimations of the stress strength parameter *R* can be further studied by considering the progressively type-II censored samples, hybrid censored samples, and estimation of *R* based on records values.

## Figures and Tables

**Table 1:** Bias, mean square error (MSE), average length of confidence interval (ALCI) with their coverage probability (CP) of various estimators of *R*.

$\alpha_{1}$	$\alpha_{2}$	$\lambda_{1}$	$\lambda_{2}$	*R*		$\left(n_{1},n_{2}\right)$				$\left(n_{1},n_{2}\right)$				$\left(n_{1},n_{2}\right)$				$\left(n_{1},n_{2}\right)$		
2.0	1.9	3.7	3.8	0.4683		(20,20)				(40,30)				(40,50)				(60,60)		
						Bias		MSE		Bias		MSE		Bias		MSE		Bias		MSE
					$R_{MLE}$	0.0021		0.0084		−0.0024		0.0049		0.0003		0.0036		0.0032		0.0029
					$R_{SEL}$	0.0140		0.0031		0.0124		0.0024		0.0045		0.0020		0.0090		0.0019
					$R_{MAP}$	0.0130		0.0039		0.0128		0.0029		0.0034		0.0023		0.0086		0.0022
					$R_{AEL}$	0.0136		0.0032		0.0124		0.0024		0.0042		0.0020		0.0089		0.0019
					$R_{LIN}$	0.0083		0.0029		0.0086		0.0023		0.0013		0.0020		0.0066		0.0019
					$R_{GEL}$	−0.0123		0.0033		−0.0048		0.0024		−0.0098		0.0022		−0.0018		0.0019
						ALCI		CP		ALCI		CP		ALCI		CP		ALCI		CP
					$AS_{CI}$	0.8396		0.90		0.3819		0.93		0.2391		0.90		0.1812		0.89
					${B_{p}}_{CI}$	0.3476		0.94		0.2669		0.94		0.2349		0.96		0.2024		0.93
					${B_{t}}_{CI}$	0.3476		0.93		0.2669		0.94		0.2349		0.95		0.2024		0.93
					$HPD_{CI}$	0.2904		0.98		0.2382		0.98		0.2167		0.98		0.1909		0.95
1.5	4.0	2.0	1.9	0.8248		(20,20)				(40,30)				(40,50)				(60,60)		
						Bias		MSE		Bias		MSE		Bias		MSE		Bias		MSE
					$R_{MLE}$	−0.0050		0.0047		−0.0034		0.0024		0.0022		0.0020		−0.0024		0.0016
					$R_{SEL}$	−0.0750		0.0075		−0.0571		0.0045		−0.0470		0.0032		−0.0438		0.0028
					$R_{MAP}$	−0.0632		0.0061		−0.0507		0.0039		−0.0434		0.0030		−0.0407		0.0027
					$R_{AEL}$	−0.0709		0.0069		−0.0548		0.0042		−0.0455		0.0031		−0.0428		0.0027
					$R_{LIN}$	−0.0785		0.0081		−0.0593		0.0048		−0.0486		0.0033		−0.0451		0.0027
					$R_{GEL}$	−0.0852		0.0094		−0.0628		0.0053		−0.0512		0.0036		−0.0472		0.0031
						ALCI		CP		ALCI		CP		ALCI		CP		ALCI		CP
					$AS_{CI}$	0.5684		0.90		0.3748		0.90		0.1762		0.89		0.1341		0.88
					${B_{p}}_{CI}$	0.2555		0.96		0.1908		0.97		0.1804		0.97		0.1536		0.96
					${B_{t}}_{CI}$	0.2555		0.96		0.1908		0.96		0.1804		0.96		0.1536		0.95
					$HPD_{CI}$	0.2217		0.83		0.1732		0.90		0.1530		0.88		0.1374		0.89
1.5	1.5	3.0	3.5	0.4690		(20,20)				(40,30)				(40,50)				(60,60)		
						Bias		MSE		Bias		MSE		Bias		MSE		Bias		MSE
					$R_{MLE}$	0.0003		0.0087		0.0005		0.0049		−0.0005		0.0037		0.0017		0.0027
					$R_{SEL}$	0.0137		0.0034		0.0132		0.0026		0.0035		0.0022		0.0064		0.0018
					$R_{MAP}$	0.0136		0.0043		0.0140		0.0032		0.0023		0.0026		0.0066		0.0021
					$R_{AEL}$	0.0135		0.0035		0.0132		0.0026		0.0031		0.0022		0.0062		0.0019
					$R_{LIN}$	0.0080		0.0032		0.0094		0.0025		0.0004		0.0022		0.0039		0.0018
					$R_{GEL}$	−0.0126		0.0034		−0.0037		0.0026		−0.0105		0.0024		−0.0044		0.0019
						ALCI		CP		ALCI		CP		ALCI		CP		ALCI		CP
					$AS_{CI}$	0.6148		0.95		0.3373		0.90		0.2060		0.90		0.1583		0.89
					${B_{p}}_{CI}$	0.3459		0.93		0.2659		0.95		0.2351		0.94		0.2024		0.96
					${B_{t}}_{CI}$	0.3459		0.92		0.2660		0.94		0.2351		0.93		0.2024		0.96
					$HPD_{CI}$	0.2905		0.98		0.2356		0.98		0.2143		0.97		0.1905		0.98
1.9	1.7	4	3.0	0.5055		(20,20)				(40,30)				(40,50)				(60,60)		
						Bias		MSE		Bias		MSE		Bias		MSE		Bias		MSE
					$R_{MLE}$	0.0029		0.0083		−0.0013		0.0051		−0.0018		0.0035		0.0005		0.0027
					$R_{SEL}$	−0.0057		0.0029		−0.0008		0.0024		−0.0068		0.0020		0.0008		0.0018
					$R_{MAP}$	−0.0048		0.0039		−0.0003		0.0029		−0.0068		0.0023		0.0006		0.0021
					$R_{AEL}$	−0.0058		0.0031		−0.0006		0.0024		−0.0069		0.0020		0.0009		0.0018
					$R_{LIN}$	−0.0115		0.0030		−0.0047		0.0024		−0.0099		0.0020		−0.0017		0.0018
					$R_{GEL}$	−0.0312		0.0042		−0.0172		0.0028		−0.0200		0.0024		−0.0094		0.0019
						ALCI		CP		ALCI		CP		ALCI		CP		ALCI		CP
					$AS_{CI}$	0.5754		0.92		0.2823		0.87		0.1868		0.89		0.1406		0.90
					${B_{p}}_{CI}$	0.3527		0.94		0.2711		0.94		0.2353		0.95		0.2034		0.95
					${B_{t}}_{CI}$	0.3527		0.94		0.2712		0.93		0.2353		0.95		0.2034		0.96
					$HPD_{CI}$	0.2907		0.99		0.2385		0.98		0.2144		0.99		0.1908		0.98
2.5	2.7	1.6	1.9	0.5068		(20,20)				(40,30)				(40,50)				(60,60)		
						Bias		MSE		Bias		MSE		Bias		MSE		Bias		MSE
					$R_{MLE}$	−0.0029		0.0023		0.0013		0.0020		0.0005		0.0019		0.0004		0.0017
					$R_{SEL}$	0.0028		0.0034		−0.0012		0.0023		0.0042		0.0021		0.0047		0.0016
					$R_{MAP}$	0.0034		0.0045		−0.0006		0.0029		0.0045		0.0024		0.0044		0.0019
					$R_{AEL}$	0.0030		0.0036		−0.0010		0.0024		0.0042		0.0021		0.0047		0.0017
					$R_{LIN}$	−0.0030		0.0035		−0.0048		0.0023		0.0013		0.0020		0.0024		0.0016
					$R_{GEL}$	−0.0220		0.0043		−0.0162		0.0027		−0.0076		0.0022		−0.0044		0.0017
						ALCI		CP		ALCI		CP		ALCI		CP		ALCI		CP
					$AS_{CI}$	0.5153		0.91		0.1734		0.80		0.1111		0.81		0.1101		0.85
					${B_{p}}_{CI}$	0.2246		0.89		0.2646		0.96		0.2341		0.95		0.2019		0.95
					${B_{t}}_{CI}$	0.2246		0.96		0.2646		0.95		0.2341		0.94		0.2019		0.95
					$HPD_{CI}$	0.2896		0.98		0.2283		0.98		0.2053		0.98		0.1807		0.98
0.9	0.9	0.9	0.9	0.5000		(20,20)				(40,30)				(40,50)				(60,60)		
						Bias		MSE		Bias		MSE		Bias		MSE		Bias		MSE
					$R_{MLE}$	0.0024		0.0079		0.0021		0.0046		−0.0002		0.0037		0.0007		0.0024
					$R_{SEL}$	0.0023		0.0048		0.0020		0.0030		−0.0001		0.0026		0.0001		0.0017
					$R_{MAP}$	0.0017		0.0061		0.0026		0.0036		−0.0004		0.0031		−0.0001		0.0021
					$R_{AEL}$	0.0023		0.0051		−0.0024		0.0031		−0.0002		0.0027		0.0002		0.0018
					$R_{LIN}$	−0.0034		0.0049		−0.0016		0.0030		−0.0028		0.0027		−0.0021		0.0017
					$R_{GEL}$	−0.0228		0.0058		−0.0133		0.0033		−0.0118		0.0029		−0.0090		0.0019
						ALCI		CP		ALCI		CP		ALCI		CP		ALCI		CP
					$AS_{CI}$	4.7021		0.69		0.1147		0.70		0.0687		0.69		0.0511		0.70
					${B_{p}}_{CI}$	0.3457		0.93		0.2631		0.95		0.2321		0.94		0.2007		0.95
					${B_{t}}_{CI}$	0.3457		0.93		0.2631		0.94		0.2321		0.93		0.2007		0.95
					$HPD_{CI}$	0.2877		0.95		0.2287		0.96		0.2032		0.94		0.1785		0.95

**Table 2:** Bias, MSE, ALCI with their CP of various estimators of *R*.

$\alpha_{1}$	$\alpha_{2}$	$\lambda_{1}$	$\lambda_{2}$	*R*		$\left(n_{1},n_{2}\right)$				$\left(n_{1},n_{2}\right)$				$\left(n_{1},n_{2}\right)$				$\left(n_{1},n_{2}\right)$		
1.7	3.5	2.0	2.1	0.7529		(20,20)				(40,30)				(40,50)				(60,60)		
						Bias		MSE		Bias		MSE		Bias		MSE		Bias		MSE
					$R_{MLE}$	−0.0021		0.0065		0.0009		0.0034		−0.0244		0.0028		−0.0007		0.0020
					$R_{SEL}$	−0.0643		0.0065		−0.0511		0.0042		−0.0454		0.0033		−0.0384		0.0027
					$R_{MAP}$	−0.0549		0.0060		−0.0454		0.00396		−0.0418		0.0032		−0.0036		0.0026
					$R_{AEL}$	−0.0601		0.0061		−0.0489		0.0040		−0.0441		0.0032		−0.0374		0.0026
					$R_{LIN}$	−0.0688		0.0072		−0.0538		0.0045		−0.0475		0.0036		−0.0400		0.0028
					$R_{GEL}$	−0.0784		0.0089		−0.0592		0.00523		−0.0516		0.0040		−0.0432		0.0031
						ALCI		CP		ALCI		CP		ALCI		CP		ALCI		CP
					$AS_{CI}$	0.5512		0.92		0.2947		0.91		0.1801		0.89		0.1361		0.82
					${B_{p}}_{CI}$	0.2937		0.94		0.2211		0.94		0.2061		0.95		0.1745		0.95
					${B_{t}}_{CI}$	0.2937		0.93		0.2211		0.94		0.2061		0.95		0.1745		0.95
					$HPD_{CI}$	0.2507		0.92		0.1983		0.89		0.1768		0.89		0.1565		0.88
2.0	2.0	2.0	1.5	0.5378		(20,20)				(40,30)				(40,50)				(60,60)		
						Bias		MSE		Bias		MSE		Bias		MSE		Bias		MSE
					$R_{MLE}$	−0.0029		0.0086		0.0010		0.0049		−0.0023		0.0037		−0.0005		0.0030
					$R_{SEL}$	−0.0174		0.0042		−0.0143		0.0028		−0.0110		0.0021		−0.0092		0.0018
					$R_{MAP}$	−0.0161		0.0051		−0.0013		0.0032		−0.0110		0.0024		−0.0089		0.0021
					$R_{AEL}$	−0.0169		0.0045		−0.0139		0.0028		−0.0108		0.0021		−0.0090		0.0018
					$R_{LIN}$	−0.0232		0.0044		−0.0179		0.0029		−0.0138		0.0021		−0.0115		0.0019
					$R_{GEL}$	−0.0418		0.0061		−0.0290		0.0036		−0.0223		0.0025		−0.0180		0.0021
						ALCI		CP		ALCI		CP		ALCI		CP		ALCI		CP
					$AS_{CI}$	0.2800		0.79		0.1556		0.78		0.0985		0.75		0.0743		0.72
					${B_{p}}_{CI}$	0.3488		0.93		0.2683		0.94		0.2357		0.96		0.2039		0.95
					${B_{t}}_{CI}$	0.3488		0.92		0.2683		0.94		0.2357		0.95		0.2039		0.94
					$HPD_{CI}$	0.2895		0.96		0.2299		0.96		0.2049		0.97		0.1814		0.96
1.7	1.8	2.5	2.5	0.5245		(20,20)				(40,30)				(40,50)				(60,60)		
						Bias		MSE		Bias		MSE		Bias		MSE		Bias		MSE
					$R_{MLE}$	0.0048		0.0083		0.0001		0.0050		0.0022		0.0038		0.0008		0.0025
					$R_{SEL}$	−0.0057		0.0034		−0.0065		0.0025		−0.0049		0.0021		−0.0036		0.0016
					$R_{MAP}$	−0.0040		0.0045		−0.0051		0.0031		−0049		0.0025		−0.0029		0.0019
					$R_{AEL}$	−0.0053		0.0035		−0.0061		0.0026		−0.0048		0.0021		−0.0034		0.0016
					$R_{LIN}$	−0.0114		0.0035		−0.0101		0.0026		−0.0079		0.0021		−0.0059		0.0016
					$R_{GEL}$	−0.0270		0.0038		−0.0216		0.0031		−0.0170		0.0025		−0.0131		0.0018
						ALCI		CP		ALCI		CP		ALCI		CP		ALCI		CP
					$AS_{CI}$	0.4264		0.85		0.2357		0.82		0.1479		0.83		0.1168		0.83
					${B_{p}}_{CI}$	0.3500		0.93		0.2681		0.93		0.2367		0.94		0.2044		0.96
					${B_{t}}_{CI}$	0.3500		0.92		0.2681		0.93		0.2367		0.94		0.2044		0.96
					$HPD_{CI}$	0.2888		0.98		0.2326		0.98		0.2100		0.98		0.1863		0.98
1.9	1.8	3.6	4.5	0.4228		(20,20)				(40,30)				(40,50)				(60,60)		
						Bias		MSE		Bias		MSE		Bias		MSE		Bias		MSE
					$R_{MLE}$	−0.0001		0.0077		0.0013		0.0046		−0.0018		0.0034		0.0002		0.0027
					$R_{SEL}$	0.0342		0.0037		0.0307		0.0067		0.0133		0.0021		0.0145		0.0020
					$R_{MAP}$	0.0321		0.0044		0.0290		0.0036		0.0111		0.0025		0.0130		0.0022
					$R_{AEL}$	0.0335		0.0038		0.0302		0.0032		0.0126		0.0021		0.0140		0.0020
					$R_{LIN}$	0.0284		0.0034		0.0268		0.0029		0.0101		0.0020		0.0120		0.0019
					$R_{GEL}$	0.0064		0.0029		0.0124		0.0025		−0.0020		0.0021		0.0027		0.0019
						ALCI		CP		ALCI		CP		ALCI		CP		ALCI		CP
					$AS_{CI}$	0.8269		0.97		0.4540		0.96		0.2306		0.94		0.2143		0.90
					${B_{p}}_{CI}$	0.3393		0.94		0.2603		0.94		0.2306		0.95		0.1990		0.94
					${B_{t}}_{CI}$	0.3393		0.93		0.2603		0.93		0.2306		0.94		0.1990		0.94
					$HPD_{CI}$	0.2903		0.98		0.2385		0.97		0.2163		0.98		0.1909		0.96
4.0	3.5	5.0	2.9	0.5489		(20,20)				(40,30)				(40,50)				(60,60)		
						Bias		MSE		Bias		MSE		Bias		MSE		Bias		MSE
					$R_{MLE}$	−0.0005		0.8882		0.0004		0.0053		0.0028		0.0037		0.0045		0.0027
					$R_{SEL}$	−0.0345		0.0036		−0.0216		0.0026		−0.0157		0.0021		−0.0047		0.0017
					$R_{MAP}$	−0.0346		0.0044		−0.0203		0.0031		−0.0155		0.0024		−0.0039		0.0020
					$R_{AEL}$	−0.0342		0.0037		−0.0213		0.0027		−0.0156		0.0021		−0.0043		0.0017
					$R_{LIN}$	−0.0402		0.0040		−0.0254		0.0028		−0.0189		0.0022		−0.0071		0.0017
					$R_{GEL}$	−0.0589		0.0061		−0.0370		0.0037		−0.028		0.0027		−0.0140		0.0019
						ALCI		CP		ALCI		CP		ALCI		CP		ALCI		CP
					$AS_{CI}$	0.5480		0.94		0.3087		0.90		0.1907		0.88		0.1429		0.80
					${B_{p}}_{CI}$	0.3536		0.95		0.2722		0.94		0.2335		0.95		0.2036		0.95
					${B_{t}}_{CI}$	0.3536		0.94		0.2722		0.93		0.2335		0.95		0.2036		0.94
					$HPD_{CI}$	0.2889		0.98		0.2374		0.98		0.2126		0.96		0.1889		0.98

**Table 3:** Estimated parameters by MLE and Bayes estimation, log-likelihood (L) and Kolmogorov–Smirnov (KS) with the *p*-values for the real data study 1.

	$\alpha_{1}$	$\alpha_{2}$	$\lambda_{1}$	$\lambda_{2}$	*L*
MLE	2.2424	2.1369	8.3180	4.5606	−109.05
Bayes	1.9001	1.7690	5.0526	2.6749	−
	$Data1_{MLE}$	$Data2_{MLE}$	$Data1_{Bayes}$	$Data2_{Bayes}$	
KS	0.0762	0.0829	0.1158	0.0942	
*p*-value	0.7894	0.7478	0.3089	0.5975	

**Table 4:** Estimated value of *R*, confidence intervals and the length of confidence interval for the real data study 1.

	$R_{\mathrm{MLE}}$	$R_{\mathrm{SEL}}$	$R_{\mathrm{MAP}}$	$R_{\mathrm{AEL}}$	$R_{\mathrm{LIN}}$	$R_{\mathrm{GEL}}$
R	0.6133	0.59654	0.61173	0.59798	0.59432	0.58887
	AS	Bp	$B_{t}$	HPD		
CI	(0.4790,0.7477)	(0.5203,0.7084)	(0.5203,0.7064)	(0.5018,0.6857)		
LCI	0.2687	0.1861	0.1861	0.1839		

**Table 5:** Estimated parameters by MLE and Bayes estimation, log-likelihood (L) and KS with the *p*-values for real data study 2.

	$\alpha_{1}$	$\alpha_{2}$	$\lambda_{1}$	$\lambda_{2}$	*L*
MLE	0.1932	0.2369	1.5734	0.3897	−489.34
Bayes	0.1722	0.2425	0.8863	0.4738	−
	$Data1_{MLE}$	$Data2_{MLE}$	$Data1_{Bayes}$	$Data2_{Bayes}$	
KS	0.0594	0.0700	0.0793	0.0718	
*p*-value	0.8724	0.9101	0.5551	0.8946	

**Table 6:** Estimated value of *R*, confidence intervals and the length of confidence interval for real data study 2.

	$R_{\mathrm{MLE}}$	$R_{\mathrm{SEL}}$	$R_{\mathrm{MAP}}$	$R_{\mathrm{AEL}}$	$R_{\mathrm{LIN}}$	$R_{\mathrm{GEL}}$
R	0.65995	0.63970	0.65208	0.64078	0.63809	0.63455
	AS	Bp	$B_{t}$	HPD		
CI	(0.6380,0.6817)	(0.5729,0.7417)	(0.5729,0.7470)	(0.5569,0.7148)		
LCI	0.0439	0.1742	0.1742	0.1579		

## Abbreviations

The following abbreviations and some notations are used in this manuscript:

AEL	absolute error loss function
ALCI	average length of confidence interval
$AS_{CI}$	asymptotic confidence interval
BE	Bayes estimation
$B_{p}$	percentile bootstrap confidence interval
$B_{t}$	student’s bootstrap confidence interval
CI	confidence interval
CP	coverage probability
GEL	general entropy loss function
HPD	highest posterior density
I(θ)	Fisher information matrix
KS	Kolmogorov-Smirnov
L	log-likelihood
LINEX	linear exponential loss function
MAP	maximum a posteriori
MLE	maximum likelihood estimation
MSE	mean square error
PHLD	Poisson half logistic distribution
R	stress-strength parameter
SEL	square error loss function

## Appendix A

The elements of J(θ):

$$\begin{array}{cc} \frac{\partial^{2}L}{\partial \alpha_{1}^{2}} & =-\frac{n_{1}}{\alpha_{1}^{2}}-2\sum_{i=1}^{n_{1}}\frac{x_{i}^{2}\;e^{-\alpha_{1}x_{i}}}{1+e^{-\alpha_{1}x_{i}}}+2\sum_{i=1}^{n_{1}}\frac{x_{i}^{2}\;e^{-2\alpha_{1}x_{i}}}{{\left(1+e^{-\alpha_{1}x_{i}}\right)}^{2}}-2\lambda_{1}\sum_{i=1}^{n_{1}}\frac{x_{i}^{2}\;e^{-\alpha_{1}x_{i}}}{{\left(1+e^{-\alpha_{1}x_{i}}\right)}^{2}}\\ \; & +4\lambda_{1}\sum_{i=1}^{n_{1}}\frac{x_{i}^{2}\;e^{-2\alpha_{1}x_{i}}}{{\left(1+e^{-\alpha_{1}x_{i}}\right)}^{3}},\\ \frac{\partial^{2}L}{\partial \alpha_{2}^{2}} & =-\frac{n_{2}}{\alpha_{2}^{2}}-2\sum_{j=1}^{n_{2}}\frac{y_{j}^{2}\;e^{-\alpha_{2}y_{j}}}{1+e^{-\alpha_{2}y_{j}}}+2\sum_{j=1}^{n_{2}}\frac{y_{j}^{2}\;e^{-2\alpha_{2}y_{j}}}{{\left(1+e^{-\alpha_{2}y_{j}}\right)}^{2}}-2\lambda_{2}\sum_{j=1}^{n_{2}}\frac{y_{j}^{2}\;e^{-\alpha_{2}y_{j}}}{{\left(1+e^{-\alpha_{2}y_{j}}\right)}^{2}}\\ \; & +4\lambda_{2}\sum_{j=1}^{n_{2}}\frac{y_{j}^{2}\;e^{-2\alpha_{2}y_{j}}}{{\left(1+e^{-\alpha_{2}y_{j}}\right)}^{3}},\\ \frac{\partial^{2}L}{\partial \lambda_{1}^{2}} & =-\frac{n_{1}}{\lambda_{1}^{2}}-\frac{n_{1}\;e^{\lambda_{1}}}{{\left(e^{\lambda_{1}}-1\right)}^{2}},\;\;\frac{\partial^{2}L}{\partial \lambda_{2}^{2}}=-\frac{n_{2}}{\lambda_{2}^{2}}-\frac{n_{2}\;e^{\lambda_{2}}}{{\left(e^{\lambda_{2}}-1\right)}^{2}},\;\;\frac{\partial^{2}L}{\partial \alpha_{1}\partial \lambda_{1}}=2\sum_{i=1}^{n_{1}}\frac{x_{i}\;e^{-\alpha_{1}x_{i}}}{{\left(1+e^{-\alpha_{1}x_{i}}\right)}^{2}}\\ \frac{\partial^{2}L}{\partial \alpha_{2}\partial \lambda_{2}} & =2\sum_{j=1}^{n_{2}}\frac{y_{j}\;e^{-\alpha_{2}y_{j}}}{{\left(1+e^{-\alpha_{2}y_{j}}\right)}^{2}},\;\;\frac{\partial^{2}L}{\partial \alpha_{1}\partial \alpha_{2}}=\frac{\partial^{2}L}{\partial \alpha_{2}\partial \alpha_{1}}=0,\;\;\frac{\partial^{2}L}{\partial \alpha_{1}\partial \lambda_{2}}=\frac{\partial^{2}L}{\partial \lambda_{2}\partial \alpha_{1}}=0\\ \frac{\partial^{2}L}{\partial \alpha_{2}\partial \lambda_{1}} & =\frac{\partial^{2}L}{\partial \lambda_{1}\partial \alpha_{2}}=0,\;\;\frac{\partial^{2}L}{\partial \lambda_{1}\partial \lambda_{2}}=\frac{\partial^{2}L}{\partial \lambda_{2}\partial \lambda_{1}}=0. \end{array}$$

The elements of I(θ), and the rest of the elements follow similar way:

$$\begin{array}{cc} \frac{\partial^{2}L}{\partial \alpha_{1}^{2}} & =-\frac{n_{1}}{\alpha_{1}^{2}}-2\sum_{i=1}^{n_{1}}E\left[\frac{x_{i}^{2}\;e^{-\alpha_{1}x_{i}}}{1+e^{-\alpha_{1}x_{i}}}\right]+2\sum_{i=1}^{n_{1}}E\left[\frac{x_{i}^{2}\;e^{-2\alpha_{1}x_{i}}}{{\left(1+e^{-\alpha_{1}x_{i}}\right)}^{2}}\right]\\ \; & -2\lambda_{1}\sum_{i=1}^{n_{1}}E\left[\frac{x_{i}^{2}\;e^{-\alpha_{1}x_{i}}}{{\left(1+e^{-\alpha_{1}x_{i}}\right)}^{2}}\right]+4\lambda_{1}\sum_{i=1}^{n_{1}}E\left[\frac{x_{i}^{2}\;e^{-2\alpha_{1}x_{i}}}{{\left(1+e^{-\alpha_{1}x_{i}}\right)}^{3}}\right]\\ \; & =-\frac{n_{1}}{\alpha_{1}^{2}}-2\sum_{i=1}^{n_{1}}\int_{0}^{\infty }\frac{x_{i}^{2}\;e^{-\alpha_{1}x_{i}}}{1+e^{-\alpha_{1}x_{i}}}f_{1}\left(x\right)dx+2\sum_{i=1}^{n_{1}}\int_{0}^{\infty }\frac{x_{i}^{2}\;e^{-2\alpha_{1}x_{i}}}{{\left(1+e^{-\alpha_{1}x_{i}}\right)}^{2}}f_{1}\left(x\right)dx\\ \; & -2\lambda_{1}\sum_{i=1}^{n_{1}}\int_{0}^{\infty }\frac{x_{i}^{2}\;e^{-\alpha_{1}x_{i}}}{{\left(1+e^{-\alpha_{1}x_{i}}\right)}^{2}}f_{1}\left(x\right)dx+4\lambda_{1}\sum_{i=1}^{n_{1}}\int_{0}^{\infty }\frac{x_{i}^{2}\;e^{-2\alpha_{1}x_{i}}}{{\left(1+e^{-\alpha_{1}x_{i}}\right)}^{3}}f_{1}\left(x\right)dx\\ \; & =-\frac{n_{1}}{\alpha_{1}^{2}}-\frac{4n_{1}\alpha_{1}\lambda_{1}}{\left(e^{\lambda_{1}}-1\right)}\int_{0}^{\infty }\frac{x^{2}\;e^{-2\alpha_{1}x+\lambda_{1}\Delta_{1}\left(x\right)}}{{\left(1+e^{-\alpha_{1}x}\right)}^{3}}dx+\frac{4n_{1}\alpha_{1}\lambda_{1}}{\left(e^{\lambda_{1}}-1\right)}\int_{0}^{\infty }\frac{x^{2}\;e^{-3\alpha_{1}x+\lambda_{1}\Delta_{1}\left(x\right)}}{{\left(1+e^{-\alpha_{1}x}\right)}^{4}}dx\\ \; & -\frac{4n_{1}\alpha_{1}\lambda_{1}^{2}}{\left(e^{\lambda_{1}}-1\right)}\int_{0}^{\infty }\frac{x^{2}\;e^{-2\alpha_{1}x+\lambda_{1}\Delta_{1}\left(x\right)}}{{\left(1+e^{-\alpha_{1}x}\right)}^{4}}dx+\frac{8n_{1}\alpha_{1}\lambda_{1}^{2}}{\left(e^{\lambda_{1}}-1\right)}\int_{0}^{\infty }\frac{x^{2}\;e^{-3\alpha_{1}x+\lambda_{1}\Delta_{1}\left(x\right)}}{{\left(1+e^{-\alpha_{1}x}\right)}^{5}}dx\\ \; & =-\frac{n_{1}}{\alpha_{1}^{2}}-\frac{4n_{1}\alpha_{1}\lambda_{1}}{\left(e^{\lambda_{1}}-1\right)}\nabla \left(\alpha_{1},2,2,3,\lambda_{1}\right)+\frac{4n_{1}\alpha_{1}\lambda_{1}}{\left(e^{\lambda_{1}}-1\right)}\nabla \left(\alpha_{1},2,3,4,\lambda_{1}\right)\\ \; & -\frac{4n_{1}\alpha_{1}\lambda_{1}^{2}}{\left(e^{\lambda_{1}}-1\right)}\nabla \left(\alpha_{1},2,2,4,\lambda_{1}\right)+\frac{8n_{1}\alpha_{1}\lambda_{1}^{2}}{\left(e^{\lambda_{1}}-1\right)}\nabla \left(\alpha_{1},2,3,5,\lambda_{1}\right). \end{array}$$

## References

[1] Wolfe D.A., Hogg R.V. (1971). On constructing statistics and reporting data. Am. Stat..

[2] Lloyd D.K., Lipow M. (1962). Reliability, Management, Methods and Mathematics.

[3] Guttman I., Johnson R.A., Bhattacharyya G.K., Reiser B. (1988). Confidence limits for stress-strength models with explanatory variables. Technometrics.

[4] Kotz S., Lumelskii Y., Pensky M. (2003). The Stress–Strength Model and Its Generalizations: Theory and Applications.

[5] Ratnam R.R.L., Rosaiah K., Anjaneyulu M.S.R. (2000). Estimation of reliability in multicomponent stress-strength model: Half logistic distribution. IAPQR Trans..

[6] Kim D.H., Kang S.G., Cho J.S. (2000). Noninformative priors for stress-strength system in the Burr-type X model. J. Korean Stat. Soc..

[7] Guo H., Krishnamoorthy K. (2004). New approximate inferential methods for the relia- bility parameter in a stress-strength model: The normal case. Commun. Stat. Theory Methods.

[8] Barbiero A. (2011). Confidence intervals for reliability of stress-strength models in the normal case. Commun. Stat. Simul. Comput..

[9] Gupta R.C., Brown N. (2001). Reliability studies of the skew-normal distribution and its application to a strength-stress model. Commun. Stat. Theory Methods.

[10] Azzalini A., Chiogna M. (2004). Some results on the stress-strength model for skew-normal variates. Metron.

[11] Shawky A.I., El Sayed H.S., Nassar M.M. (2001). On stress-strength reliability model in generalized gamma case. IAPQR Trans..

[12] Khan M.A., Islam H.M. (2009). On strength reliability for generalized gamma distributed stress. J. Stat. Theory Appl..

[13] Nadarajah S. (2004). Reliability for logistic distributions. Elektron. Model..

[14] Asgharzadeh A., Valiollahi R., Raqab M.Z. (2013). Estimation of the stress-strength reliability for the generalized logistic distribution. Stat. Meth..

[15] Nadarajah S. (2004). Reliability for Laplace distributions. Math. Prob. Eng..

[16] Kundu D., Gupta R.D. (2005). Estimation of P(Y < X) for generalized exponential distribution. Metrika.

[17] Krishnamoorthy K., Mukherjee S., Guo H. (2007). Inference on reliability in two- parameter exponential stress-strength model. Metrika.

[18] Baklizi A. (2014). Interval estimation of the stress-strength reliability in the two-parameter exponential distribution based on records. J. Stat. Comput. Simul..

[19] Baklizi A., El-Masri A.Q. (2004). Shrinkage estimation of *P*(*X*<*Y*) in the exponential case with common location parameter. Metrika.

[20] Kayid M., Elbatal I., Merovci F. (2016). A new family of generalized quadratic hazard rate distribution with applications. J. Test. Eval..

[21] Abbas K., Tang Y. (2014). Objective Bayesian analysis of the Fréchet stress-strength model. Stat. Prob. Lett..

[22] Ghitany M.E., Al-Mutairi D.K., Aboukhamseen S.M. (2015). Estimation of the reliability of a stress-strength system from power Lindley distributions. Commun. Stat. Simul. Comput..

[23] Okasha H.M., Kayid M., Abouammoh M.A., Elbatal I. (2016). A new family of quadratic hazard rate-geometric distributions with reliability applications. J. Test. Eval..

[24] Nadarajah S., Bagheri S., Alizadeh M., Samani E. (2018). Estimation of the Stress Strength Parameter for the Generalized Exponential-Poisson Distribution. J. Test. Eval..

[25] Nadarajah S. (2005). Reliability for some bivariate beta distributions. Math. Prob. Eng..

[26] Shrahili M., Elbatal I., Muhammad I., Muhammad M. (2020). Properties and applications of beta Erlang-truncated exponential distribution. J. Math. Comput. Sci. JM.

[27] Muhammad M., Lixia L. (2019). A New Extension of the Generalized Half Logistic Distribution with Applications to Real Data. Entropy.

[28] Ahmad K.E., Jaheen Z.F., Yousef M.M. (2010). Inference on Pareto distribution as stress- strength model based on generalized order statistics. J. Appl. Stat. Sci..

[29] Krishnamoorthy K., Lin Y. (2010). Confidence limits for stress-strength reliability involv- ing Weibull models. J. Stat. Plan. Infer..

[30] Kundu D., Gupta R.D. (2006). Estimation of P[Y < X] for Weibull distributions. IEEE Trans. Reliab..

[31] Asgharzadeh A., Valiollahi R., Raqab M.Z. (2011). Stress-strength reliability of Weibull distribution based on progressively censored samples. SORT.

[32] Asgharzadeh A., Kazemi M., Kundu D. (2015). Estimation of *P*(*X*<*Y*) for Weibull distribution based on hybrid censored samples. Int. J. Syst. Assur. Eng. Manag..

[33] Valiollahi R., Asgharzadeh A., Raqab M.Z. (2013). Estimation of *P*(*Y*<*X*) for Weibull distribution under progressive type-II censoring. Commun. Stat. Theory Methods.

[34] Asgharzadeh A., Valiollahi R., Raqab M.Z. (2017). Estimation of *P**r*(*Y*<*X*) for the two- parameter generalized exponential records. Commun. Stat. Simul. Comput..

[35] Kohansal A. (2019). Bayesian and classical estimation of *R*=*P*(*X*<*Y*) based on Burr type XII distribution under hybrid progressive censored samples. Commun. Stat. Theory Methods.

[36] Yadav A.S., Singh S.K., Singh U. (2019). Bayesian estimation of stress–strength reliability for Lomax distribution under type-II hybrid censored data using asymmetric loss function. Life Cycle Reliab. Saf. Eng..

[37] Alaa H., Properties A.-H. (2016). Estimations and Predictions for a Poisson- Half-Logistic Distribution Based on Progressively Type-II Censored Samples. Appl. Math. Model..

[38] Canuto C., Hussaini M.Y., Quarteroni A., Zang T.A. (2006). Spectral Methods: Fundamentals in Single Domains.

[39] Muhammad M., Yahaya M.A. (2017). The Half Logistic-Poisson Distribution. Asian J. Math. Appl..

[40] Muhammad M. (2017). Generalized Half Logistic Poisson Distributions. Commun. Stat. Appl. Methods.

[41] Efron B., Tibshirani R.J. (1993). An Introduction to Bootstrap.

[42] Calabria R., Pulcini G. (1996). Point estimation under asymmetric loss function for left truncated exponential samples. Commun. Stat. Theory Meth..

[43] Metropolis N., Rosenbluth A., Rosenbluth M., Teller A., Teller E. (1953). Equations of state calculations by fast computing machine. J. Chem. Phys..

[44] Hastings W.K. (1970). Monte Carlo sampling methods using Markov chains and their appli- cations. Biometrika.

[45] Gelfand A.E., Smith A.F.M. (1990). Sampling-based approaches to calculating marginal densities. J. Am. Stat. Assoc..

[46] Chen M.H., Shao Q.M. (1999). Monte Carlo estimation of Bayesian Credible and HPD intervals. J. Comput. Graph. Stat..

[47] Meredith M., Kruschke J. (2018). HDInterval: Highest (Posterior) Density Intervals. R Package Version 0.2.0. https://CRAN.R-project.org/package=HDInterval.

[48] R Core Team (2019). R: A Language and Environment for Statistical Computing.

[49] Al-Mutairi D.K., Ghitany M.E., Kundu D. (2015). Inferences on stress-strength reliability from weighted lindley distributions. Commun. Stat. Theory Meth..

[50] Badar M.G., Priest A.M., Hayashi T., Kawata K., Umekawa S. (1982). Statistical aspects of fibre and bundle strength in hybrid composites. Progress in Science and Engineering Composites.

[51] Raqab M.Z., Kundu D. (2005). Comparison of different estimators of *P*(*Y*<*X*) for a scaled Burr Type X distribution. Commun. Stat. Simul. Comput..

[52] Surles J.G., Padgett W.J. (1998). Inference for Reliability and Stress-Strength for a Scaled Burr-Type X Distribution. Lifetime Data Anal..

[53] Surles J.G., Padgett W.J. (2001). Inference for *P*(*Y*<*X*) in the Burr-Type X Model. J. Appl. Stat. Sci..

[54] Al-Mutairi D.K., Ghitany M.E., Kundu D. (2013). Inferences on stress-strength reliability from lindley distributions. Commun. Stat. Theory Meth..

[55] Ali S. (2013). On the mean residual life function and stress and strength analysis under different loss function for lindley distribution. J. Qual. Reliab. Eng..

[56] Singh S.K., Singh U., Sharma V.K. (2014). Estimation on system reliabilityin generalized Lindley stress-strength model. J. Stat. Appl. Prob..

[57] Sadek A. (2018). Mostafa Mohie Eldin and Shaimaa Elmeghawry. Estimation of Stress-Strength Reliability for Quasi Lindley Distribution. Adv. Syst. Sci. Appl..

[58] Lindley D.V. (1958). Fiducial distributions and bayes theorem. J. R. Stat. Soc..

